# A new water-soluble silicon phthalocyanine that catalyzes the photodegradation of pollutant dyes

**DOI:** 10.55730/1300-0527.3715

**Published:** 2024-12-21

**Authors:** Damla Nur KAYA, Behice Şebnem SESALAN

**Affiliations:** Department of Chemistry, Faculty of Science and Letters, İstanbul Technical University, İstanbul, Turkiye

**Keywords:** Phthalocyanines, dyes, photocatalysis, first-order UV-vis spectroscopy

## Abstract

The removal of color arising from water-soluble dyes in wastewaters is necessary to counter the threat to health. Because of the difficulty in elimination of these dyes by conventional methods, their photodegradation using photosensitizers such as phthalocyanines (Pcs) has been employed recently. When compared to peripherally substituted derivatives, silicon Pcs are reported to be more biocompatible with low toxicity. Thus a new silicon Pc substituted with two quaternized dimethylamino phenoxy units (compound 1) and its quaternized derivative (compound 2) were synthesized to test its photocatalytic ability employing the dyes methylene blue (MB), eosin B (EB), erythrosine (ERB), sulforhodamine B (SRB), and brilliant blue FCF (BRB), some of whose absorption bands overlap with those of Pcs. The overlapped absorption bands were split using first-order derivative UV-vis spectra. The photodegradation rates of BRB, MB, and ERB were 41%, 38%, and 29%, respectively, under 30-s short time irradiation in the presence of compound **2**. The plots of the natural logarithm of the concentrations of dyes versus time fit the first-order reaction model. According to the experimental data, compound **2** could be used as a photocatalyst due to singlet oxygen generation for photodegradation of pollutant dyes MB, ERB, and BRB with higher photodegradation rates.

## 1. Introduction

Nowadays, textile and industrial dyes are becoming one of the most threatening types of chemicals because of their growing pollution of waters. Dyes pose a serious global environmental problem as their reinforced structures make them stable to photodegradation under severe conditions [1].

Dyes as well as flavors, sweeteners, antioxidants, and antimicrobial preservatives could exhibit toxicity when exceeding the permitted dose. For example, brilliant blue FCF (BRB), used as a coloring agent in food, can induce malignant tumors, asthma, and hyperactivity [2].

To date, dyes that are harmful to human health have mainly come from the textile industry. Even a very small amount of coloring substance makes it stable to light, heat and oxidizing agents. For instance, while eosin B (EB) is used extensively in printing [3], another cationic dye, methylene blue (MB), is extensively applied to cotton and silk as a colorant, resulting in eye burns or in some cases permanent injury to the eyes. Moreover, previous studies have confirmed that MB may lead to serious problems in the nervous system such as mental confusion, breathing problems, and methemoglobinemia [4]. A water-soluble cherry-pink anionic xanthene dye, erythrosine B (ERB), serves as a potential colorant in textiles such as wool, silk, and nylon and in a variety of food items. Allergic reactions in the eyes, irritation of skin and the upper respiratory tract, severe headaches, and nausea [5] were reported as the hazardous effects of ERB. Another study about the harmful aspects of sulforhodamine B (SRB), which is a pollutant dye used for leather, was presented by Li et al., who examined its photooxidation [6].

Recently, phthalocyanines (Pcs) have emerged as a potential chemical class for many applications in different fields and have gained much attention owing to their unique nature, whose properties can be tailored according to different demands [7]. The substitution of functional groups either on the periphery of Pc rings [8] or axially [9–11] improves their optical, catalytic, and electronic properties and enables them to be used in different medical and biological applications [12,13] such as gas sensors, catalysts [14], nonlinear optical materials [15], solar cells [16], photosensitizers in photodynamic therapy [17], and for remediation of wastewater [18].

To deal with the problem of contamination of water, water soluble Pcs are one of the most beneficial macrocycles regarding their thermally and chemically stable structures, which can act as photocatalysts. As reported in the literature, Pcs, especially axially substituted silicon derivatives, have good solubility, biocompatibility, high singlet oxygen quantum yield, and low dark toxicity [19]. According to this aspect, axially substituted silicon Pcs became very popular in biological studies since the first axially substituted silicon Pc, named Pc4, was prepared and approved for clinical trials with attractive properties such as very low molecular aggregation in solution, low dark toxicity, and fast clearance from the body when used in photodynamic therapy [20]. Thus silicon Pcs have been focused on to deal with medicinal problems such as cancer after the approval of Pc4 in clinics.

The first reason for using the dimethylamino group in the SiPc ring was its ease to quaternize. Secondly, dimethylamino groups are one of the most frequently used classes of functional group in drug chemistry. In the body, amino groups are converted to ammonium cations and form salt bridges with other anionic units. Thus these ionic interactions occur easily with drugs that contain amino groups and are the main factors for drug designs to correspond to diseases directly [21].

These interactions are the main factors for drug design to respond correctly to the disease.

While silicon Pc derivatives have been used more widely in biomedical applications, substituted peripheral metallo Pcs have been utilized in the photodegradation of dyes in water treatment. For instance, rhodamine dye was subjected to photodegradation using copper Pc hollow spheres [22]. Musial et al. examined nanocomposites of titanium dioxide and peripherally substituted Pcs on photodegradation of dyes for the remediation of wastewaters, tested many metallophthalocyanines with different peripheral groups and central metal ions, and compared the results [23]. Another study was presented on the photodegradation of methyl orange and orange G with the photocatalytic activity of asymmetrical and symmetrical zinc phthalocyanine–cobalt ferrite conjugates [24]. A nanocomposite made up of copper Pc derivative–titanium nanopowder was employed for photodegradation of MB under irradiation as presented by Cabir et al. [25].

Herein, attention should be given to the emerging uses of silicon Pcs mainly in photodynamic therapy after approval of Pc4 for clinical studies [20] rather than remediation of wastewaters such as photodegradation of pollutants. A second particular aspect to motivate our objective was that generally peripherally substituted metallo Pcs were involved for photocatalytic applications [26–28] in previous studies. In the light of this literature, we engaged in obtaining a water soluble silicon Pc derivative (compound **2**) for the photodegradation of dyes in water. Another critical point was that the maximum absorption bands of the common selected dyes that were subjected to degradation with Pcs did not overlap with characteristic Q or B bands of Pcs derivatives in previously reported papers [29,30]. The maximum absorptions of some pollutant dyes are noted in the Q-band region of Pc. Thus, an adequate selection of dyes to be photodegraded was the last critical step of our work. The dyes MB, EB, ERB, SRB, and BRB, some of whose absorption bands overlap with the Q-band region of the new compound **2**, were employed to examine photodegradation. First-order derivative UV-vis spectroscopy was beneficial for monitoring the split of overlapped bands and the recognition and the stability of the Q band. Further, the singlet oxygen and photodegradation quantum yields supported the generation of singlet oxygen causing decreases in the absorption maxima of dyes.

The kinetics of photodegradation was fitted to the first-order model. The dye removal rates were also calculated and BRB was the most rapidly decomposed dye at a degree of 41% among them, followed by MB and ERB with 39% and 29%, respectively. The least degraded dye was SRB with 9% after 30-s irradiation. According to the results, the photocatalytic effect of compound **2** without any nanoparticle support was noteworthy.

## 2. Materials and methods

^1^H NMR spectra were recorded using a Varian Mercury 500 MHz spectrometer in DMSO-d_6_. Chemical shifts were reported relative to Me4Si as an internal standard. Mass spectra were measured on a Micromass Quatro LC/ULTIMA LC–MS/MS spectrometer. Elemental analyses were performed with Thermo Finnigan Flash EA 1112 at 950–1000 °C. Elemental analysis and mass spectra were obtained from the Instrumental Analysis Laboratory of TÜBİTAK. Absorbance measurements were recorded using a Shimadzu UV-1800 UV spectrophotometer with 1-cm path-length quartz cuvettes in the spectral range of 300–800 nm. Photoirradiation was done using a General Electric quartz line halogen lamp (300 W). A 600-nm glass cut-off filter (Schott) and a water filter were used to filter off ultraviolet and infrared radiation, respectively. An interference filter (Intor, 670 nm with a band width of 40 nm) was additionally placed in the light path before the sample. Light intensities were measured with a THORLABS power meter. All dyes, silicon phthalocyanine dichloride, and 1,3-diisobenzofuran (DPBF) were purchased from Sigma Aldrich and 9,10-antracenediyl-bis(methylene)dimalonoic acid (ADMA) were purchased from Fluka. UV-vis measurements were done in 3.5-mL quartz cuvettes. To see the differences in absorption bands of the aforementioned dyes in free form (given in Figures S1–S6 in the Supplementary Figures) and after addition of compound **2** to the solutions (Figures S7–S11 in the Supplementary Figures), zero- and first-order derivative UV-vis spectroscopy was used.

### 2.1. Bis[(3-N,N-dimethylamino)-phenoxy]-phthalocyaninato-silicon(IV) (1, C_48_H_36_N_10_O_2_Si)

Silicon(IV) phthalocyanine dichloride (50 mg, 0.082 mmol) was reacted with dimethylaminophenol (53 mg, 0.389 mmol) in the presence of sodium hydride (12 mg, 0.5 mmol) in 10 mL of dry toluene. The reaction mixture was refluxed at 110 °C for 10 h. The dark green mixture was centrifuged for 10 min and the filtrate was collected. Then the solvent was removed under pressure and dried in vacuo. The obtained dry product was dissolved in THF and subsequently purified by silica gel column chromatography in which CH_3_OH and CHCl_3_ (0.1:5) were employed as eluent. Yield: 60 mg (9.76%). ^1^H NMR (500 MHz, DMSO-d_6_): δ = 10.06–9.12 (m, 8H, Hα); 8.78–7.97 (m, 8H, Hβ); 6.64–6.08 (m, 8H, Ar-H), 3.23–2.71 (m, 12H, N-CH_3_) ppm (Figure S12 in the Supplementary Figures); UV-vis (THF, 4.82 × 10^−5^ mol dm^−3^); λmax (ɛ) = 353 (549.54), 610 (208.92), 676 (1023.29) (Figure S13 in Supplementary Figures). MALDI-TOF m/z = 812.27 [M]^+^; found: 1122.116 [M+2DHB]^+^ (matrix used in MALDI is DHB. DHB = 3,5-dihydroxybenzene) (Figure S14 in the Supplementary Figures). Elemental analysis calcd (%) for C_48_H_36_N_10_O_2_Si: C 70.92; H 4.46; N 17.23; found: C 71.07; H 3.98; N 18.04.

### 2.2. Quaternized bis[(3-N,N-dimethylamino)-phenoxy]-phthalocyaninato-silicon(IV) (2, C50H42I2N10O2Si)

Synthesized compound **1** (66 mg, 81.2 μmol) and diethyl ether (3 mL) were stirred with excess methyl iodide at room temperature for 24 h in the dark. At the end of the 24 h, the green mixture was filtered and the precipitate was collected and then dried in vacuo. Yield: 40 mg (6.32%). ^1^H NMR (500 MHz, DMSO-d_6_) δ = 9.74–9.47 (m, 8H, Hα); 8.51–8.26 (m, 8H, Hβ); 7.18–6.91 (m, 8H, Ar-H), 2.93–2.91 (m, 18H, N-CH_3_) ppm (Figure S15 in the Supplementary Figures); UV-vis (H_2_O, 5.70 × 10^−4^ mol dm^−3^); λmax (ɛ) = 351 (407.38), 621 (165.95), 690 (1976.96) (Figure S6 in the Supplementary Figures). MALDI-TOF m/z = 1098.37 [M]^+^ (Figure S16 in the Supplementary Figures). Elemental analysis calcd (%) for C_50_H_42_I_2_N_10_O_2_Si: C 54.74; H 3.86; N 12.77; found: C 56.06; H 2.98; N 13.08.

### 2.3. Singlet oxygen quantum yields of compounds 1 and 2

Photoirradiation was done using a 300-W halogen lamp. A dichroic filter (Shimadzu) was additionally placed in the light path before the sample. Then 3 mL of 1 × 10^−5^ M compound **1** containing the singlet oxygen quencher 1,3-diisobenzofuran (DPBF) was irradiated in the Q-band region with the photoirradiation setup given in section 1 as ref. [1] in the Supplementary Figures.

### 2.4. Photodegradation quantum yields of compounds 1 and 2

To determine the stability of compounds **1** and **2**, photodegradation quantum yields (Φ_d_) were determined using the experimental setup and calculations described in previously published papers [31–36]. Details are given in the Supplementary Figures in section 2.

### 2.5. UV-vis measurements

**Figure 1:**
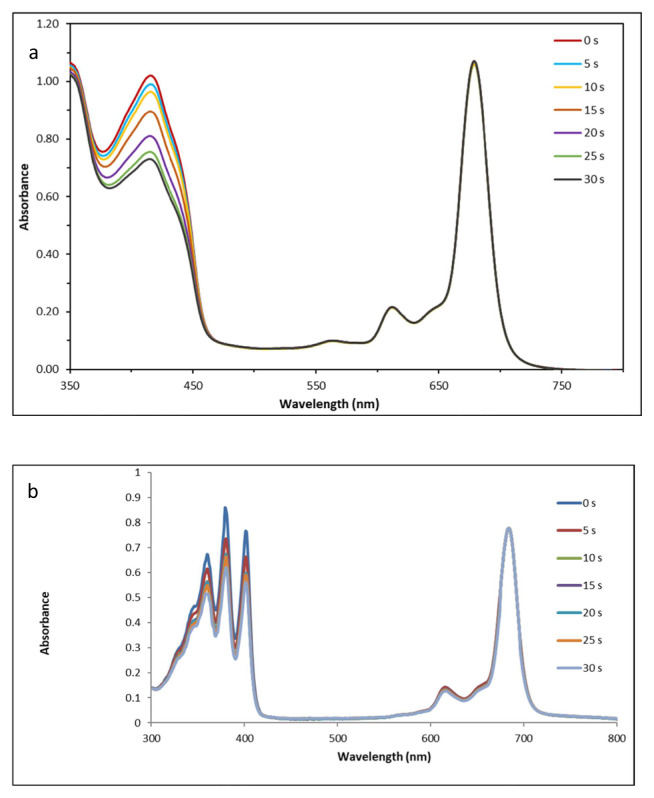
The electronic spectrum showing the decreases in absorbance of DPBF at 417 nm (a) for compound **1** and ADMA at 380 nm (b) for compound **2** after 30-s irradiation.

**Figure 2:**
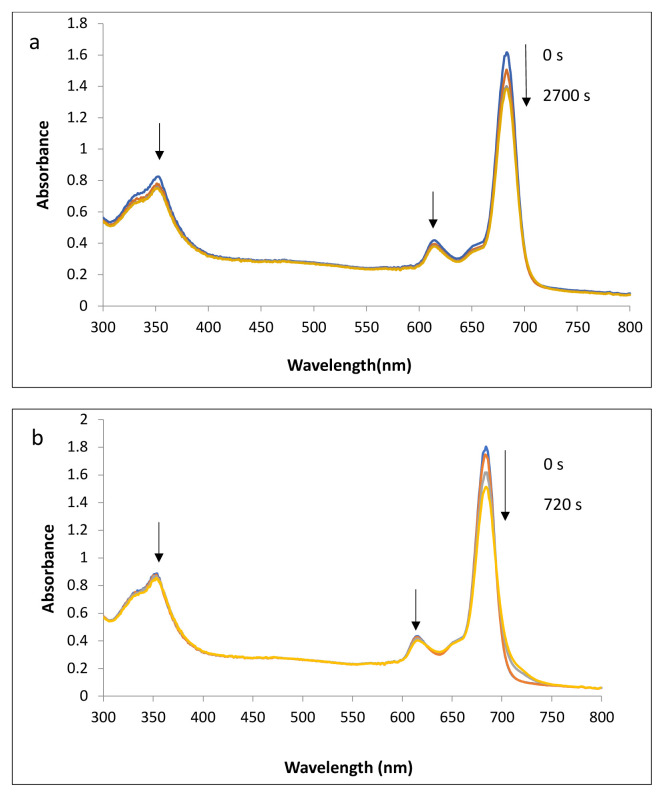
The electronic spectra showing the photodegradation of compound **1** in DMSO (a) and compound **2** in water (b) recorded after irradiation.

UV-vis measurements were obtained in 3.5-mL quartz cuvettes. The characteristic absorption bands of the aforementioned dyes in free form shown in Supplementary Figures S1–S6 and UV-vis spectra were recorded in the absence of compound **2** at the concentrations given in Table 1. To observe the effect of irradiation on photodegradation, aqueous solutions of free dyes were prepared and irradiated for 30 s and then both zero- and first-order spectra were recorded. Later, 1.5 mL of compound **2** at a concentration of 5.70 × 10^−4^ M was mixed with 2 mL of one of the dyes at different concentrations given in Table 1 in each cuvette. First- and zero-order derivative UV-vis spectra were recorded after successive irradiation with 5-s time intervals by halogen lamp at a power of 1.5 mW in the dark. While Figures 1a and 1b indicate the singlet oxygen generation of compounds 1 and **2**, the photodegradation of these compounds is shown in Figures 2a and 2b.

### 2.6. Observation of dye–compound 2 complex formation

**Figure 3:**
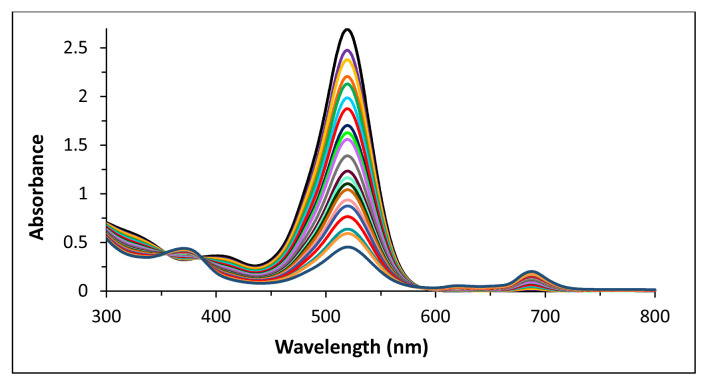
Changes in the UV-vis spectrum of EB (black line: no compound **2**) after successive additions of 6.1 × 10^−4^ M 20 μL (red line) of compound **2** up to 100 μL (dark green line at the bottom).

To evaluate the possibility of any dye–compound 2 complex formation, 6.1 × 10^−5^ M EB was titrated with 20 successive additions of 5 μL of 6.1 × 10^−4^ M compound **2** up to 100 μL (Figure 3).

## 3. Results and discussion

### 3.1. Synthesis of compounds 1 and 2

**Scheme 1:**
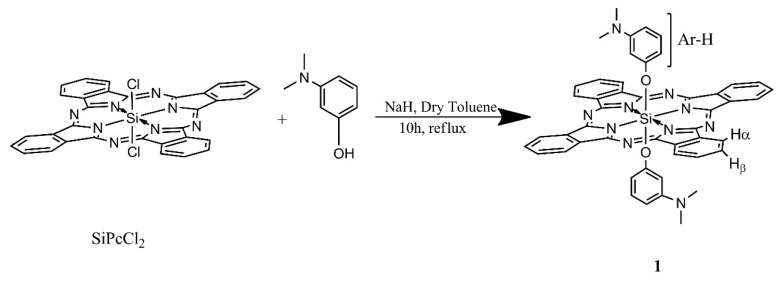
The synthetic pathway of compound **1**.

**Scheme 2:**
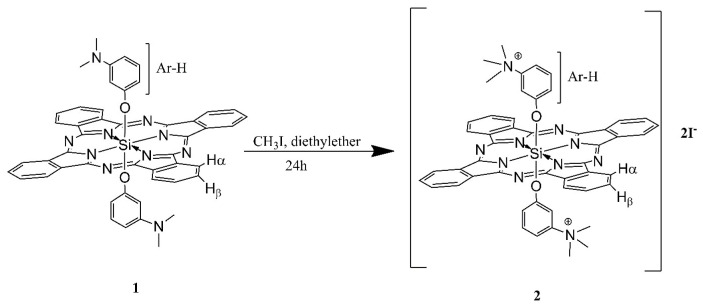
The synthetic pathway of compound **2**.

The synthetic procedures for the Pc compounds **1** and **2** are given in Schemes 1 and 2. New silicon Pcs were prepared in the presence of NaH for deprotonation of alcohol derivatives. While the silicon phthalocyanine dichloride (SiPcCl_2_) was insoluble in toluene, the new product 1 was soluble. Excess iodomethane was used in order to obtain complete quaternization. For the characterization of compounds 1 and 2, ^1^H NMR, UV-vis, MALDI-TOF mass spectra, and elemental analysis results were used.

### 3.2. Singlet oxygen quantum yields of compounds 1 and 2

The singlet oxygen quantum yield (*Φ*_Δ_) determinations were carried out using the experimental setup described in the literature [37]. The *Φ*_Δ_ value of compound 1 was calculated in DMSO by the relative method, using unsubstituted zinc(II) phthalocyanine (Std-ZnPc) as a reference. 1,3-Diphenylisobenzofuran (DPBF) was used as a chemical quencher for determination of singlet oxygen generation in DMSO. Compound 1 and Std-ZnPc (*Φ*_ΔStd_ = 0.67 in DMSO, 0.45 in water) solutions (c = 1 × 10^−5^ M) containing the singlet oxygen quencher were irradiated in the Q-band region with the photoirradiation setup described previously [37].

The diminution of DPBF absorbance at 417 nm during the light irradiation was monitored by UV-vis spectrophotometer. There was no change in the Q-band intensity of compound 1 during the light irradiation for *Φ*_Δ_ determinations, confirming the stable nature of compound 1 during the singlet oxygen study because no changes were observed in the shape or intensity of the Q band of this compound. The *Φ*_Δ_ value of compound 1 was 0.21 in DMSO (Figure 1a).

Aqueous solution of compound 2 containing 9,10-antracenediyl-bis(methylene) dimalonoic acid (ADMA) was prepared in the dark and irradiated in the Q-band region using a setup similar to that described for DPBF. The photodegradation of ADMA by compound 2 was monitored with the decreases in absorbance at 380 nm (in water). While the singlet oxygen quantum yield of the standard SiPcCl_2_ was 0.15 as reported previously [38], 0.28 was determined for compound 2 in aqueous media (Figure 1b).

### 3.3. Photodegradation quantum yields of compounds 1 and 2

Photodegradation is an oxidative degradation process a compound undergoes when exposed to irradiation and photodegradation of catalysts is vitally important for photocatalytic applications. The photodegradation rate can be determined by the photodegradation quantum yield (F_d_). Catalysts should be stable during catalysis to generate singlet oxygen. The stability of the compounds suggested as catalysts are determined with F_d_ values. As reported earlier [31,32], stable zinc Pcs have F_d_ values as low as 10^−6^ and of the order of 10^−3^ for unstable ones. The F_d_ values calculated for compounds 1 (in DMSO) and 2 (in water) were 2.15 × 10^−5^ and 0.82 × 10^−5^, respectively, indicating that quaternization of compound 1 might inhibit photoinduced electron transfer, resulting in a higher quantum yield [31,32]. There was no phototransformation during photodegradation, which was important for photocatalysts. Consequently, compounds 1 and 2 provided sufficient stability during photoirradiation at a degree of 10^−5^ (F_d_) values (Figures 2a and 2b) [31,32].

### 3.4. Titration of EB with compound 2 in the absence of irradiation

Taking into consideration the potential of the formation of a complex between selected pollutant dyes and compound 2 due to the electrostatic interactions, EB was titrated with compound 2 to observe the changes in absorption of both EB and compound 2. EB is given as an example; the other titrations with compound 2 are not included here.

While a decrease in the maximum absorption of EB was observed, small increases around the Q band of compound 2 were clear due to increasing concentration in the solution. The decrease in maximum absorption after successive additions of compound 2 could be attributed to a formation of dye–compound 2 complex (Figure 3).

Based on Figure 3, the decrease in maximum absorption of EB might indicate the potential for the formation of complexes between charged dyes and compound 2. However, since the main purpose of the present work was to test the photocatalytic effect of compound 2, aqueous solutions of dye–compound 2 were prepared and the changes in UV-vis spectra were evaluated under irradiation.

### 3.5. Examination of photodegradation of selected dyes

The changes in UV spectra were followed step by step. First, the zero-order derivative spectra of free dyes (dyes without becoming a component of a possible dye–compound 2 complex) were recorded as a control group (Figures S1–S6 in the Supplementary Figures) and then zero- and first-order derivative spectra of free dye solutions were obtained during increasing irradiations to evaluate only the effect of irradiation on photodegradation where very little decreases in absorbance were observed. The second step was the examination of photodegradation after the inclusion of the new compound 2 into aqueous solutions by means of both zero- and first-order derivative UV-vis spectra. Initial concentrations of compound 2 and dyes in each solution are given in Table 1.

#### 3.5.1. Evaluations of photodegradation of dyes

To compare the shifts of maximum absorption bands or overlap with the Q band of compound 2, we first obtained the zero-order UV-vis spectra of all dyes.

In the absence of compound 2, while the maximum absorption was noted at 663.5 nm with a shoulder at 607 nm for MB (Figure S1 in the Supplementary Figures), only one maximum for EB (Figure S2 in the Supplementary Figures) and two maxima were observed for SRB (Figure S3 in the Supplementary Figures) at 516.5 and 565.5 nm. The zero-order derivative UV-vis spectra of free ERB and BRB given in Figures S4 and S5 in the Supplementary Figures showed maxima at 530 nm and 565 nm, respectively.

Zero- (a) and first- (b) order graphics of MB, EB, SRB, ERB, and BRB free dyes given as Figures S7a and S7b, S8a and S8b, S9a and S9b, S10a and S10b, and S11a and S11b, respectively, in the Supplementary Figures indicate the resistance to photodegradation with very little decreases in the maxima under increasing irradiation.

In the case of inclusion of compound 2, all zero-order UV-vis spectra of compound 2–dye solutions indicated a probable compound 2–dye complex due to ionic interactions.

**Figure 4:**
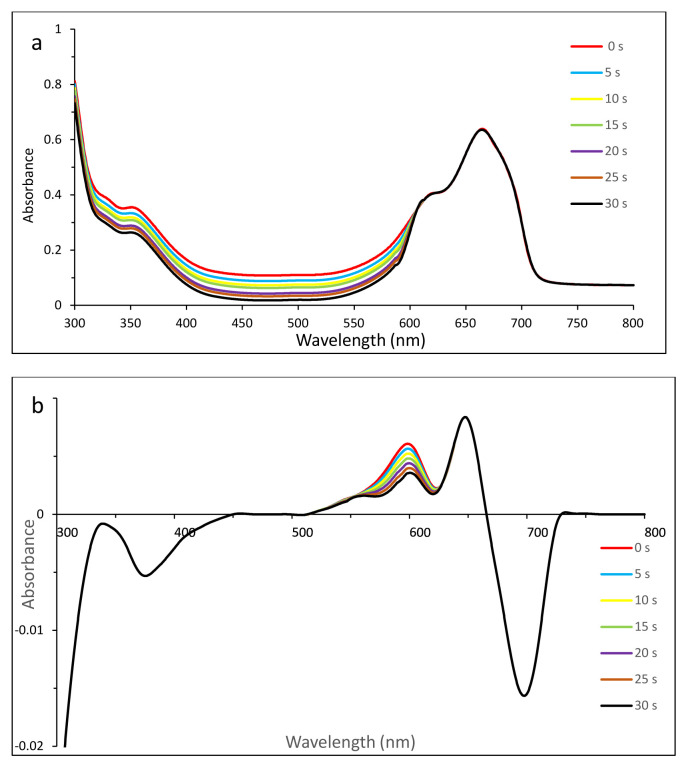
Changes in zero- (a) and first- (b) order derivative UV-vis spectrum of MB in the presence of compound **2** after irradiation with 5-s time intervals. C_0_ [MB] = 1.14 × 10^−5^ M, pH = 7.2.

Thus zero-order derivative spectra were not sufficient to show the photodegradation of dyes clearly. To split the overlapped bands and to observe the photodegradation of dyes, first-order derivative UV-vis spectra were employed (Figures 4 for MB, 5 for EB, 6 for SRB, 7 for ERB, 8 for BRB in Table 2).

#### 3.5.2. Photodegradation of MB in the presence of compound 2

While the zero-order derivative spectrum (Figure 4a) indicated that there might be an ionic interaction between compound 2 and MB, the first-order derivative spectrum (Figure 4b) showed that the photodegradation of MB in the presence of compound 2 was much more obvious due to the splitting of bands. The stable Q band of compound 2 at 690 nm was clearly observed.

All irradiation was done in the Q-band region of compound 2. Thus to observe the stability of the Q band was important for singlet oxygen generation. Compound 2 caused observable decreases in the intensities of absorbance between 572 and 626 nm due to singlet oxygen, which can be attributed to MB degradation.

#### 3.5.3. Photodegradation of EB in the presence of compound 2

**Figure 5:**
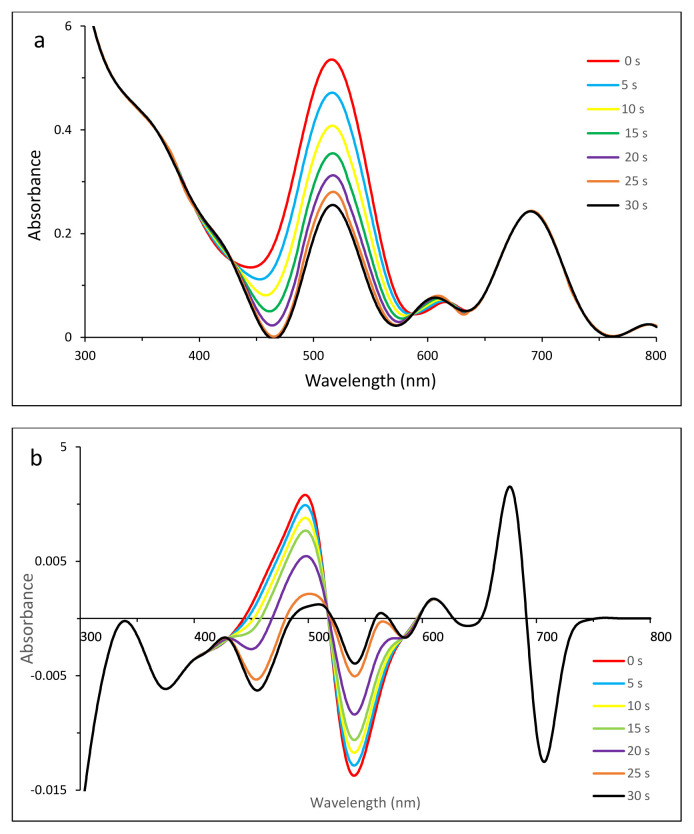
Changes in zero- (a) and first- (b) order derivative UV-vis spectrum of EB in the presence of compound **2** after irradiation with 5-s time intervals. C_0_[EB] = 2.28 × 10^−5^ M, pH = 5.2.

Due to the absorption of free EB at 516.5 nm, the bands might not overlap on each other as in the case of MB. However, it is within the realms of possibility that an ionic complex could form, resulting in changes in the zero-order derivative UV-vis spectrum (Figure 5a).

After involvement of compound 2, the first-order derivative UV-vis spectrum showed that while there was no change in Q-band absorption, the shoulder at around 620 nm of compound 2 could shift to 607 nm due to either a probable complex or photodegradation of EB (Figure 5b) after successive irradiation. However, Figure 5b proved the stability of the Q band and the decreases in the maximum around 519 nm could be assigned to photodegradation.

#### 3.5.4. Photodegradation of SRB in the presence of compound 2

**Figure 6:**
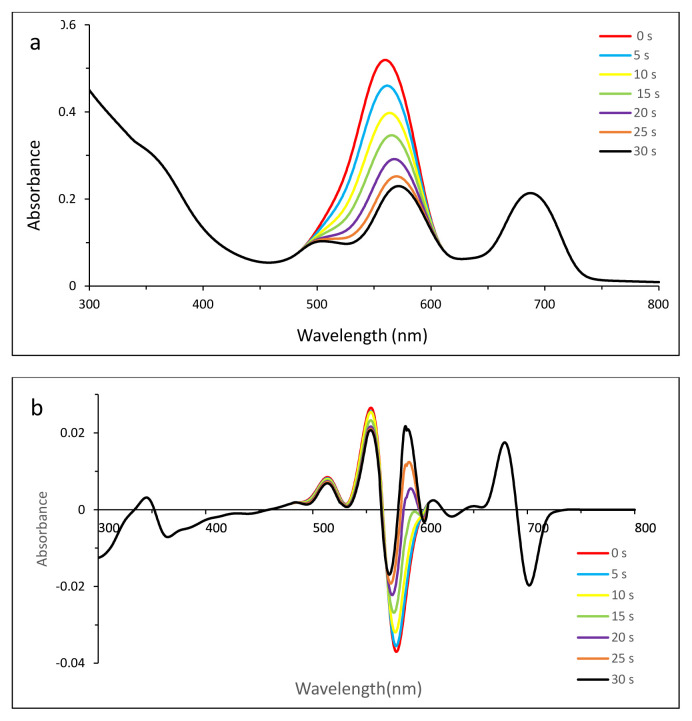
Changes in zero- (a) and first- (b) order derivative UV-vis spectrum of SRB in the presence of compound **2** after irradiation with 5-s time intervals. C_0_ [SRB] = 8.52 × 10^−5^ M, pH = 4.9.

Due to the ionic interaction with compound 2 and SRB, a tendency for complex formation could contribute to the shift of the shoulder of compound 2 at 620 nm to 611 nm. After the solution was exposed to irradiation, the changes in absorbance between 500 and 600 nm could show photodegradation due to the red shift of dye maximum from 565.5 to 578 nm (Figure 6a). For further investigation, the first-order derivative spectrum (Figure 6b) indicated that there was no decrease in intensity of the Q band around 690 nm and the shift of absorbance, confirming the photodegradation of SRB.

#### 3.5.5. Photodegradation of ERB in the presence of compound 2

**Figure 7:**
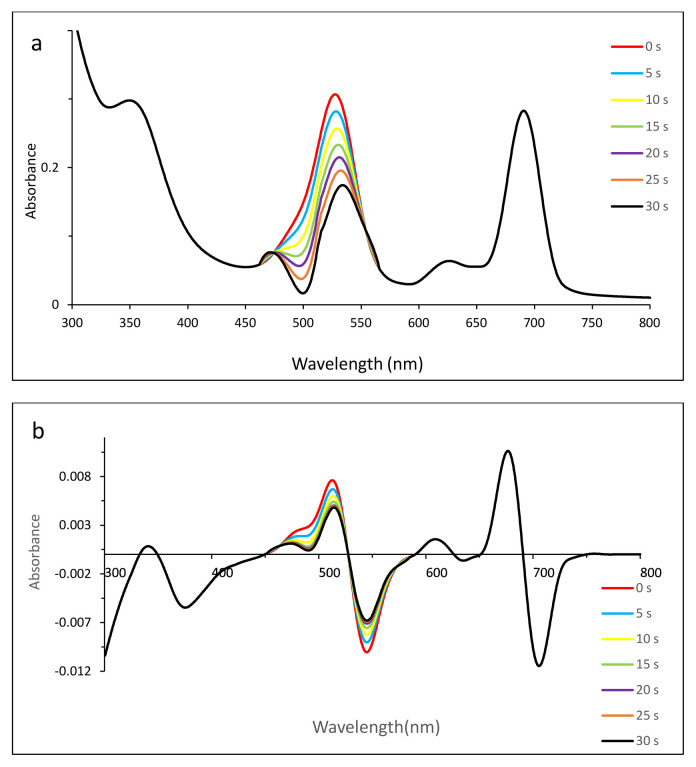
Changes in zero- (a) and first- (b) order derivative UV-vis spectrum of ERB in the presence of compound **2** after irradiation with 5-s time intervals. C_0_ [ERB] = 4.40 × 10^−5^ M, pH = 6.9.

Although Figure 7a might indicate possible ionic ERB–compound 2 complex formation, Figure 7b could show that the generation of singlet oxygen might lead to the photodegradation of ERB, which resulted in greater decreases in absorptions around 530 nm, while compound 2 was preserving its own stability seen in the Q band.

#### 3.5.6. Photodegradation of BRB in the presence of compound 2

**Figure 8:**
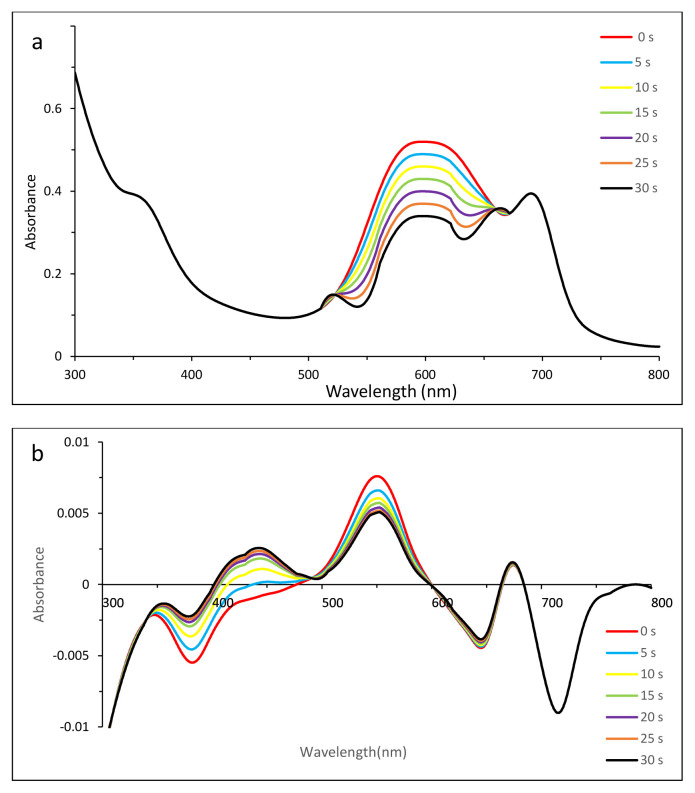
Changes in zero- (a) and first- (b) order derivative UV-vis spectrum of BRB in the presence of compound **2** after irradiation with 5-s time intervals. C_0_ [BRB] = 5.77 × 10^−5^ M, pH = 7.1.

In Figure 8a, a maximum broad absorbance at 597 nm and the blue shift of this absorbance to a broad one at 594 nm could be attributed to ionic attraction.

In Figure 8b, overlapped bands were split and the stable Q band at around 690 nm was seen clearly. Under irradiation, the decreases at between 300 and 650 nm might indicate the photodegradation of BRB due to singlet oxygen generation by compound 2.

### 3.6. The kinetics of photodegradation

**Figure 9:**
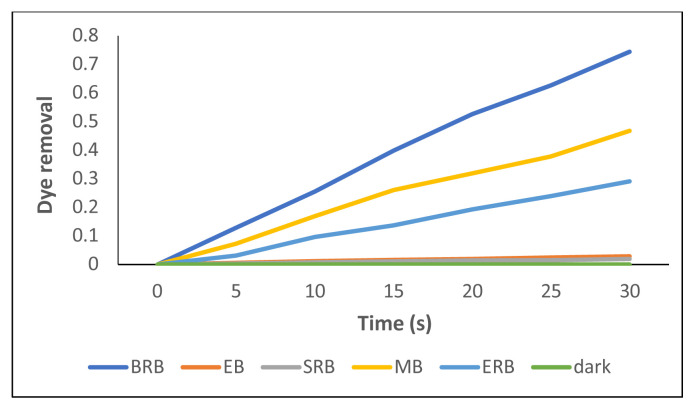
Photodegradation rates of dyes in the presence of compound **2** after 30-s irradiation.

A set of measurements in the dark and visible region light (λ = 690 nm) were employed to remove pollutant dyes from the water samples. The degradation efficiency of the as-prepared samples was defined in terms of the C/C_0_ ratio, where C and C_0_ represent the remnant and initial concentrations of dyes, respectively. Compound 2 without any support like TiO_2_ was used in the photodegradation experiments. The removal of dyes in the dark (no degradation was observed in the dark) and after 30 s irradiation with 5-s intervals is shown in Figure 9. The percentages of degradation of the dyes are given in Table 3.

BRB could be photodegraded efficiently when irradiated in the presence of compound 2 at a percentage of 40.51. MB was the second dye, whose degradation was around 38.35%. The least decomposition was seen in SRB. After the generation of an efficient singlet oxygen from compound 2, almost half of BRB was decomposed under 30-s irradiation. The results might indicate that due to ionic attraction between positively charged compound 2 and negatively charged dyes such as BRB, ERB, or EB neutral complexes formed supported with measured pH values of solutions.

In the case of MB, this cationic dye might not form a stable complex with positively charged compound 2. However, a positive formal charge localized on sulfur or nitrogen in MB could destabilize it, resulting in decomposition under irradiation. Delocalization of the negative charge on EB and SRB through COOH and SO_3_H, respectively, could make them more stable even under irradiation. Neutralization of EB and SRB with compound 2 might not occur due to charge balance. The pH values of EB and SRB supplied the information that the solutions were acidic, which makes the anions stable.

In the presence of irradiation, compound 2 was able to generate reactive oxygen species, especially singlet oxygen. According to the pH values of the solutions, the dyes such as BRB, MB, and ERB, which formed neutral complexes with compound 2, would be destabilized. Moreover, singlet oxygen produced by the Pc core could decompose these dyes more easily than those such as SRB and EB that were more stable in solution. A similar case was seen in the UV-vis spectra of DNA after the interaction of cationic Pcs [9]. Negatively charged phosphate groups (like negatively charged dyes) interact with positive Pcs and under irradiation fragments of DNA were observed in gel electrophoresis clearly. Thus the same concept might be applied for the photodegradation of dyes.

**Figure 10:**
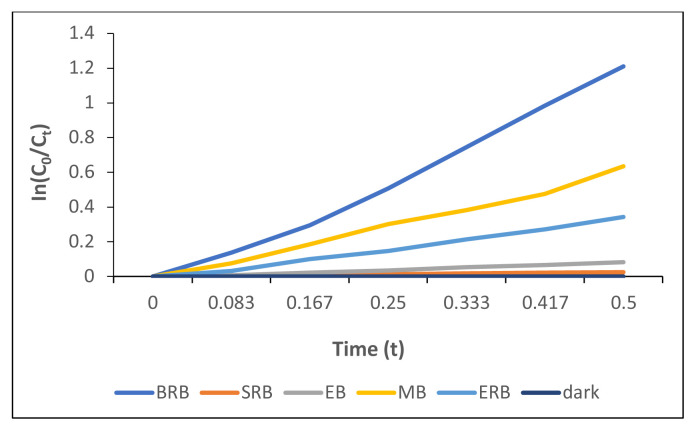
The photodegradation kinetic profile of dyes in the presence of compound **2** under irradiation.

To examine the kinetics of photodegradation, Figure 10 was integrated.

The kinetics of the first-order model was tested to determine the rate of degradation processes of MB, EB, SRB, ERB and BRB, commonly expressed by the following equation:


(1) $$\text{ln }({\text{C}}_{0}/{\text{C}}_{\text{t}})=\text{kt}$$

where k is the photodegradation rate constant (min^−1^) and C_0_ and C_t_ are the concentrations of dyes initially and after at 30-s time interval irradiations, respectively.

Both Table 3 and Figure 10 indicate that the photodegradation reactions followed first-order kinetics corresponding to the fact that with a longer irradiation time, more photodegradation of dyes occurred. The 30-s short time irradiation in the presence of compound 2 was important to observe a significant amount of photodegradation for (for instance MB, ERB, and BRB) practical use in wastewater treatment.

### 3.7. Comparison of compound 2 with other Pc catalysts

Table 4 contains a collection of previous studies about the photodegradation of dyes with metallo Pcs, which are mainly substituted with peripheral groups (the same dyes that were studied in the present work were selected from the previous reports to show which Pc composite was used for that dye). There have been a few studies that showed the catalytic effect of Pcs without support that are mainly substituted peripherally. For instance, a peripheral substituted Pc, 2(3), 9(10), 16(17), 23(24)-tetrakis-(6-methylpyridin-2-yloxy)phthalocyanine, was used to degrade orange G [29] and another study reported by Yılmaz et al. stated that Pcs bearing four carboxylic-acid-functionalized thiophenyl groups had photocatalytic effects on orange G and methyl orange [39]. Therefore, we focused on investigation of the photocatalytic ability of a water soluble silicon Pc without any support due to its more biocompatible nature together with dimethylamino units that has medical benefits due to ionic interactions [20,21] in the body.

## 4. Conclusion

Because of the ionic nature of all compounds, we considered the probability of the formation of ionic complexes between compound 2 and dyes to explain the changes in zero-order derivative UV-vis spectra when compound 2 added to the solution of free dye without irradiation. All UV-vis spectra showed completely different appearances when dyes were mixed with compound 2 without irradiation. After being titrated with compound 2, the decreases in the maxima of EB might suggest that the ionic nature of all compounds could have a dominating effect on the formations of compound 2–dye complexes (Figure 3). The titrations of other dyes with compound 2 were similar to that of EB and are not given here.

The pH values of solutions of BRB, MB, and ERB supported that neutral complexes could form and destabilize dyes while EB and SRB showed less affinity to interact with compound 2 due to more delocalized negative charge through COOH and SO_3_H, respectively, which provided them with more stability even under irradiation. Of course, to prove complex formation requires many detailed experiments and we did not concentrate on the mechanistic details of this interaction between these charged compounds. Instead, we examined the singlet oxygen generation of compound **2** that had no composite support and the photodegradation degrees of five pollutant dyes.

While the selected free dyes that were exposed to irradiation confirmed the resistance to degradation with very small decreases in maxima (Figures S7a–S11b in the Supplementary Figures), after involvement of compound 2, the greater decreases in maximum absorptions indicated the contribution of singlet oxygen to photodegradation.

The second particular point of our study was the selection of dyes, some of whose absorptions overlapped with those of characteristic Pcs. In the literature, dyes used in catalytic experiments had absorption bands that do not overlap with those of the Q-band region of Pcs [29,39]. Herein, while MB and BRB have maximum absorption at around 664 and 585 nm, respectively, a maximum at 690 nm was seen in the UV-vis spectrum of compound 2. In the solutions it was hard to distinguish overlapped absorption bands and to observe the photodegradation.

Thus, we employed the first-order derivative UV-vis spectra to distinguish the overlapped bands of compound 2 (especially Q band) and dyes. Although Pcs are identified with Q and B bands, the B-band region of water-soluble Pcs overlaps with the maxima of ADMA, which is a standard compound used to prove singlet oxygen generation with decreasing absorbance between 300 and 400 nm [37]. The stability of the Q band of Pcs is the critical step and is proved with no change in shape or intensity. Based on this fact, rather than the B band, the Q-band region was taken into consideration. For compound 2, Figure 2 was consistent with the literature [31,32,37,38] confirming singlet oxygen generation. Black lines at around 690 nm corresponding to the Q band indicated that after each 5-s irradiation, there was no change in the intensity of absorption and all irradiations were recorded on top of each other. This was the proof of the stability of the Q band. In addition, decreases in intensities of maxima between 500 and 600 nm were observed with different colors after each irradiation. Thus, the first-order derivative spectra enabled us to see the changes in the Q band and other absorption regions that dyes dominated mainly.

The singlet oxygen quantum yields of compounds 1 and 2 were 0.21 in DMSO and 0.26 in water, respectively. The ionic nature of aqueous solutions might hinder photoinduced electron transfer and result in a higher quantum yield for quaternized derivative compound 2 [31–36]. The photodegradation quantum yields for compounds 1 and 2 were calculated as 2.15 × 10^−5^ and 0.82 × 10^−5^, respectively, which were in the range of stable compounds [31,32,34–36]. Singlet oxygen quantum yields and photodegradation studies supported each other, proving that compound 2 was stable enough to be used in photocatalytic reactions owing to its stable Q band and did not degrade during irradiations, as observed in Figure 1b.

The degradation rates of the dyes are given in Table 3. According to the results, the decreasing degradation was in the order of BRB, MB, ERB, EB, and SRB. Almost half of BRB was photodegraded under 30-s short time irradiation and the plots of the natural logarithm of the concentrations of dyes versus time fit the first-order reaction model given in Figure 10. Thus the amount of dye to degrade was directly proportional with irradiation time. All the results led us to recommend compound 2, an efficient singlet oxygen generator, as a photocatalyst to remove dyes in water.

## Supplementary information

### 1. Singlet oxygen quantum yields

Singlet oxygen quantum yield (F_D_) determinations were carried out using the experimental setup described in the literature [1]. Typically, a 3-cm^3^ portion of the respective phthalocyanine derivatives (concentration = 1 × 10^−5^ M) containing the singlet oxygen quencher was irradiated in the Q-band region with the photoirradiation setup described in the references [1]. Singlet oxygen quantum yields (Φ_Δ_) were determined in air using the relative method with unsubstituted ZnPc (in DMSO) or ZnPcS_mix_ (in aqueous media) as references. DPBF and ADMA were used as chemical quenchers for singlet oxygen in DMSO and aqueous media, respectively. Eq. (1) was employed for the calculations:


(1) $$\Phi_{\Delta }=\Phi_{\Delta }^{\text{Std}}\frac{\text{R}.{\text{I}}_{\text{abs}}^{\text{Std}}}{{\text{R}}^{\text{Std}}.{\text{I}}_{\text{abs}}},$$

where ${\ddot{\text{O}}}_{\ddot{\text{A}}}^{\text{Std}}$ is the singlet oxygen quantum yields for the standard unsubstituted ZnPc ( ${\ddot{\text{O}}}_{\ddot{\text{A}}}^{\text{Std}}=0.67$ in DMSO) [2–5] and ZnPcS_mix_ ( ${\ddot{\text{O}}}_{\ddot{\text{A}}}^{\text{Std}}=0.45$ in aqueous media) [6–8]. R and R_Std_ are the DPBF (or ADMA) photobleaching rates in the presence of the phthalocyanine derivatives and standards, respectively. I_abs_ and ${\text{I}}_{\text{abs}}^{\text{Std}}$ are the rates of light absorption by phthalocyanine derivatives and standards, respectively. To avoid chain reactions induced by DPBF (or ADMA) in the presence of singlet oxygen, the concentration of quenchers (DPBF or ADMA) was lowered to ~3 × 10^−5^ M. Solutions of sensitizer containing DPBF (or ADMA) were prepared in the dark and irradiated in the Q-band region using the setup described above. DPBF degradation at 417 nm (in DMSO) and ADMA degradation at 380 nm (in water) were monitored.

### 2. Photodegradation quantum yields

Photodegradation quantum yield (Φ_d_) determinations were carried out using the experimental setup described in the literature [1,6–8]. Photodegradation quantum yields were determined using Eq. (2).


(2) $$\Phi_{\text{d}}=\frac{({\text{C}}_{0}-{\text{C}}_{\text{t}}).\text{V}.\text{NA}}{{\text{I}}_{\text{abs}}.\text{S}.\text{t}},$$

where C_0_ and C_t_ are the samples’ (compounds **1** and **2**) concentrations before and after irradiation, respectively, V is the reaction volume, N_A_ is Avogadro’s constant, S is the irradiated cell area, t is the irradiation time, and I_abs_ is the overlap integral of the radiation source light intensity and the absorption of the samples.

Supplementary References1

BrannonJH
MagdeD

Picosecond laser photophysics. Group 3A phthalocyanines
Journal of the American Chemical Society
1980
102
62
65
10.1021/ja00521a011
2

UslanC
KöksoyB
DurmuşM
Durmuş İşleyenN
ÖztürkY


The synthesis and investigation of photochemical, photophysical and biological properties of new lutetium, indium, and zinc phthalocyanines substituted with PEGME-2000 blocks
Journal of Biological Inorganic Chemistry
2019
24
191
210
10.1007/s00775-019-01638-5
30673878
3

AtmacaGY
ErdoğmuşA

Synthesis of new water soluble silicon phthalocyanine substituted by linker sulfur atom and photophysicochemical studies for photodynamic therapy
Journal of Porphyrins and Phthalocyanines
2019
23
1398
1405
10.1142/S1088424619501487
4

OgunsipeA
NyokongT

Photophysical and photochemical studies of sulphonated non-transition metal phthalocyanines in aqueous and non-aqueous media
Journal of Photochemistry and Photobiology A: Chemistry
2005
173
211
220
10.1016/j.jphotochem.2005.03.001
5

GökselM
BiyikliogluZ
DurmuşM

The water soluble axially disubstituted silicon phthalocyanines: photophysicochemical properties and in vitro studies
Journal of Biological Inorganic Chemistry
2017
22
953
967
10.1007/s00775-017-1473-0
28616663
6

Seotsanyana-MokhosiI
KuznetsovaN
NyokongTi

Photochemical studies of tetra-2,3-pyridinoporphyrazines
Journal of Photochemistry and Photobiology A: Chemistry
2001
140
215
222
10.1016/S1010-6030(01)00427-0
7

KuznetsovaNA
KaliyaOL

Heterogenized metallophthalocyanines for photodynamic microorganism inactivation: an overview of our experience
MHC
2015
8
8
19
8

WilkinsonF
HelmanWP
RossAB

Quantum yields for the photosensitized formation of the lowest electronically excited singlet state of molecular oxygen in solution
Journal of Physical and Chemical Reference Data
1993
22
113
262
10.1063/1.555934


## Supplementary Figures

UV-vis spectra of selected free dyes and compound 2 in aqueous solutions

Figure S1 1.14 × 10^−5^ M MB in aqueous solution (max. absorption at 663.5 nm).

Figure S2 2.18 × 10^−5^ M EB in aqueous solution (max. absorption at 516.5 nm).

Figure S3 8.52 × 10^−5^ M SRB in water (max. absorption at 565.5 nm).

Figure S4 4.40 × 10^−5^ M ERB in aqueous solution (max. absorption at 526 nm).

Figure S5 5.77 × 10^−5^ M BRB in aqueous solution (max. absorption at 584 nm).

Figure S6 5.70 × 10^−4^ M compound **2** (Q band at 690 nm, shoulder at 620 nm, and B band at 354 nm).

Zero (graphics a) and first order (graphics b) derivative UV-vis spectra of free dyes in aqueous solutions under irradiation with 5-s time intervals

Figure S7a Zero-order derivative spectrum of 1.14 × 10^−5^ M MB dye under 30-s irradiation.

Figure S7b First-order derivative spectrum of 1.14 × 10^−5^ M MB dye under 30-s irradiation.

Figure S8a Zero-order derivative spectrum of 2.18 × 10^−5^ M EB dye under 30-s irradiation.

Figure S8b First-order derivative spectrum of 2.18 × 10^−5^ M EB dye under 30-s irradiation.

Figure S9a Zero-order derivative spectrum of 8.52 × 10^−5^ M SRB dye under 30-s irradiation.

Figure S9b First-order derivative spectrum of 8.52 × 10^−5^ M SRB dye under 30-s irradiation.

Figure S10a Zero-order derivative spectrum of 4.41 × 10^−5^ M ERB dye under 30-s irradiation.

Figure S10b First-order derivative spectrum of 4.41 × 10^−5^ M ERB dye under 30-s irradiation.

Figure S11a Zero-order derivative spectrum of 5.77 × 10^−5^ M BRB dye under 30-s irradiation.

Figure S11b First-order derivative spectrum of 5.77 × 10^−5^ M BRB dye under 30-s irradiation.

Figure S12 ^1^H NMR spectrum of compound **1** (DMSO-d_6_).

Figure S13 UV-vis spectrum of 4.82 × 10^−5^ M compound **1** in THF (Q band at 683 nm, shoulder at 614 nm, and B band at 353 nm).

Figure S14 MALDI-TOF spectrum of compound **1**.

Figure S15 ^1^H NMR spectrum of compound **2** (DMSO-d_6_).

Figure S16 MALDI-TOF spectrum of compound **2**.

## Figures and Tables

**Table 1:** The concentrations and maximum absorption wavelengths of free dyes and compound **2** in aqueous solutions.

Dye	Concentration (M)	Wavelength (nm)
Methylene blue (MB)	1.14 × 10^−5^	663.5
Eosin B (EB)	2.18 × 10^−5^	516.5
Sulforhodamine B (SRB)	8.52 × 10^−5^	565.5
Erythrosine B (ERB)	4.41 × 10^−5^	526
Brilliant blue FCF (BRB)	5.77 × 10^−5^	585.5
Compound **2**	5.70 × 10^−4^	690

**Table 2:** UV-vis spectra of compound **2**–dye solutions.

UV spectra of compound **2**–dye solutions	Zero-order UV-vis spectrum	First-order UV-vis spectrum
compound **2**–MB	Figure 4a	Figure 4b
compound **2**–EB	Figure 5a	Figure 5b
compound **2**–SRB	Figure 6a	Figure 6b
compound **2**–ERB	Figure 7a	Figure 7b
compound **2**–BRB	Figure 8a	Figure 8b

**Table 3:** The photodegradation percentages of dyes.

Dye	Degradation (%)
MB	38
EB	11
SRB	9
ERB	29
BRB	41

**Table 4:** The dyes photodegraded by Pc composites.

Dye	Pc composite	Degradation %	Refs.
Orange G, methyl orange	Tetrathiophenycarboxyphthalocyaninato Zn(II) complex	While the degradation for orange G was 6.41% and 28.0% in the absence and presence of TX, for methylene orange it was 13.3% and 66.0% in the absence and presence of TX, respectively.	[39]
MB	Manganese Pc modified Nano-BiVO_4_	The discoloration percentages after 3 repeats were 98.49%, 98.42%, 98.41%.	[40]
MB, Eosin Y	Tetra amino zinc Pc embedded PANI sensitized Fe_2_O_3_	Optimum degradation of MB dye is obtained at pH 7 where 99% degradation was achieved in 80 min Optimum degradation of EY dye is obtained at pH 4 where 94.4% degradation was achieved in 80 min.	[41]
Rhodamine B	Iron Pc supported on amidoximated PAN fiber	Amidoximated PAN fibers (AO-PAN). catalyst 1: AO-PAN, CP% = 60.6%, CFe = 0.068 mmol/g could remove nearly 100% of rhodamine B	[42]
Acid Red 1 (AR1), Reactive Red 2 (RR2), Reactive Red 24 (RR24), Reactive (RB221) Direct Red 31 (DR31)	Fiber supported cobalt Pc	In the absence of H_2_O_2_: RR2 (after 30 min) 79.86, RR24 (after 60 min) 81.39, RR195 (after 60 min) 78.78, RB221 (after 30 min) 80.88, DR31 (after 3 min) 50.52%.	[43]
Methyl orange	ZnPc supported on Fe_2_O_3_	Degradation rate constant: 0.0078/min.	[44]
Rhodamine B, MB	Core-shell Ni–Co spinel coated with FePc	Degradation rate for rhodamine B: 79.9% conversion/60 min, 99% conversion/60 min of MB.	[44]
