# Supplementary figures and images for: A new water-soluble silicon phthalocyanine that catalyzes the photodegradation of pollutant dyes
Source: Turk J Chem. 2024 Dec 21;49(1):118–32. doi: 10.55730/1300-0527.3715 (PMC11913358; doi:10.55730/1300-0527.3715)

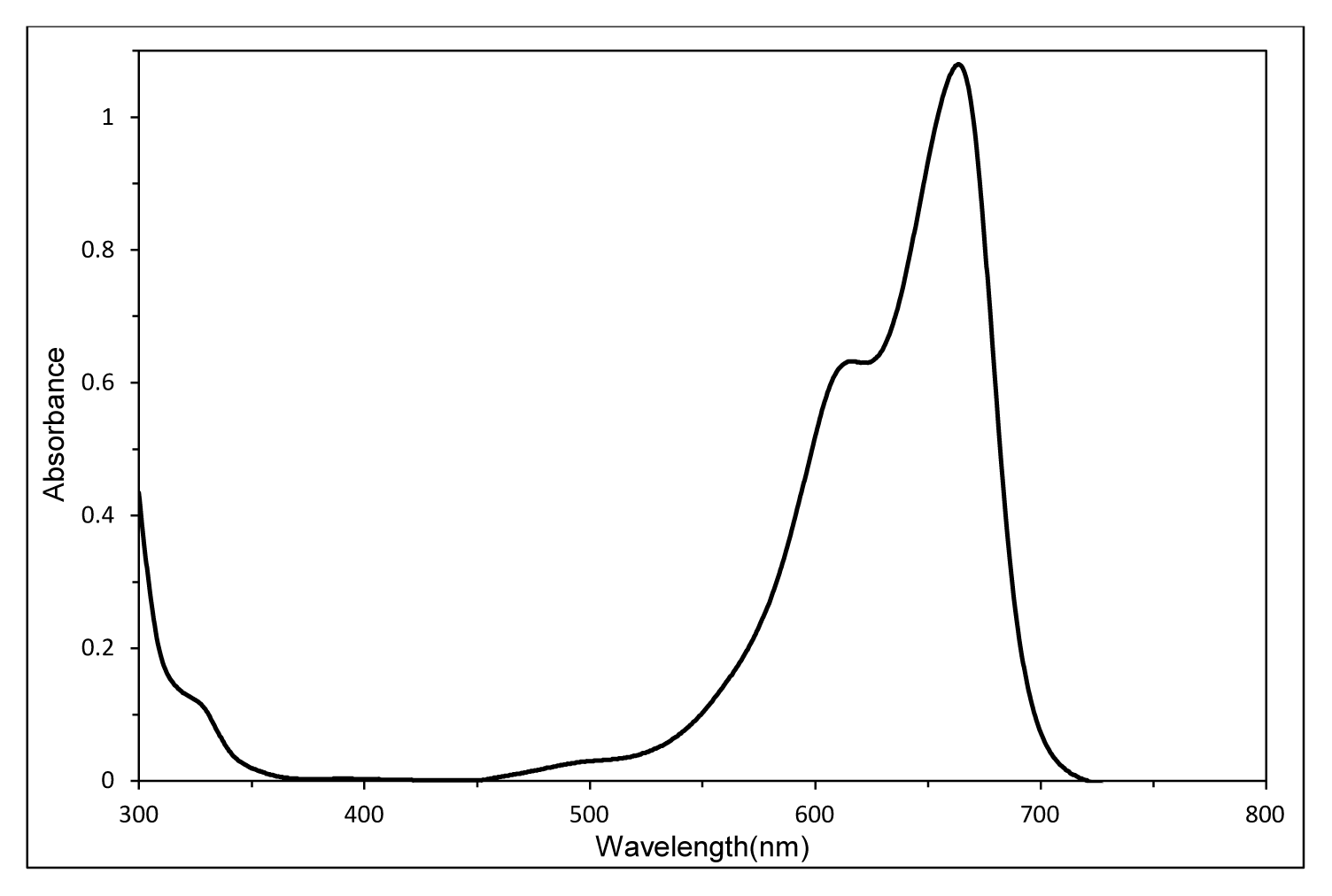

Supplement: Figure S1 — 1.14 × 10^−5^ M MB in aqueous solution (max. absorption at 663.5 nm). [file tjc-49-01-118s1.tif]

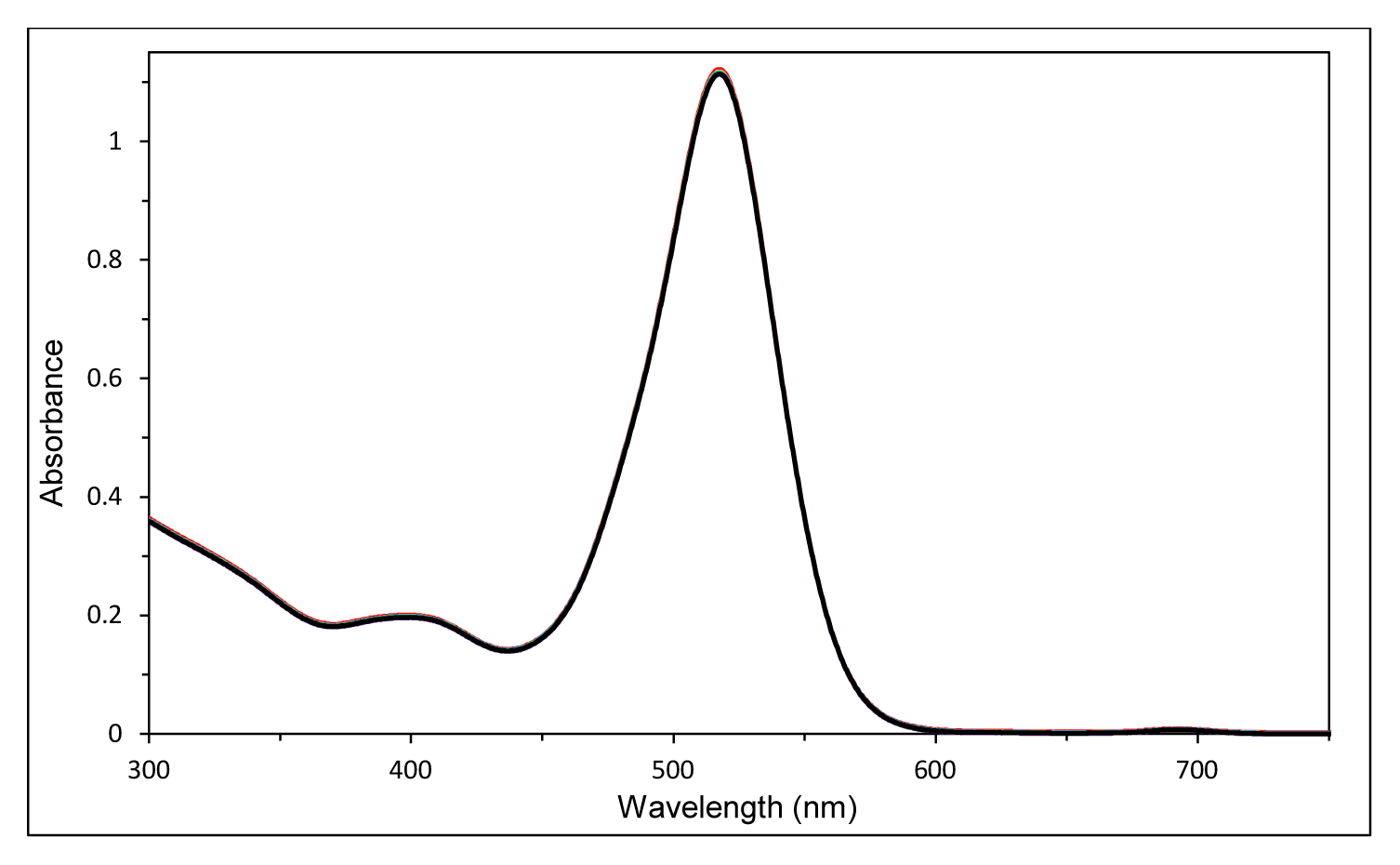

Supplement: Figure S2 — 2.18 × 10^−5^ M EB in aqueous solution (max. absorption at 516.5 nm). [file tjc-49-01-118s2.tif]

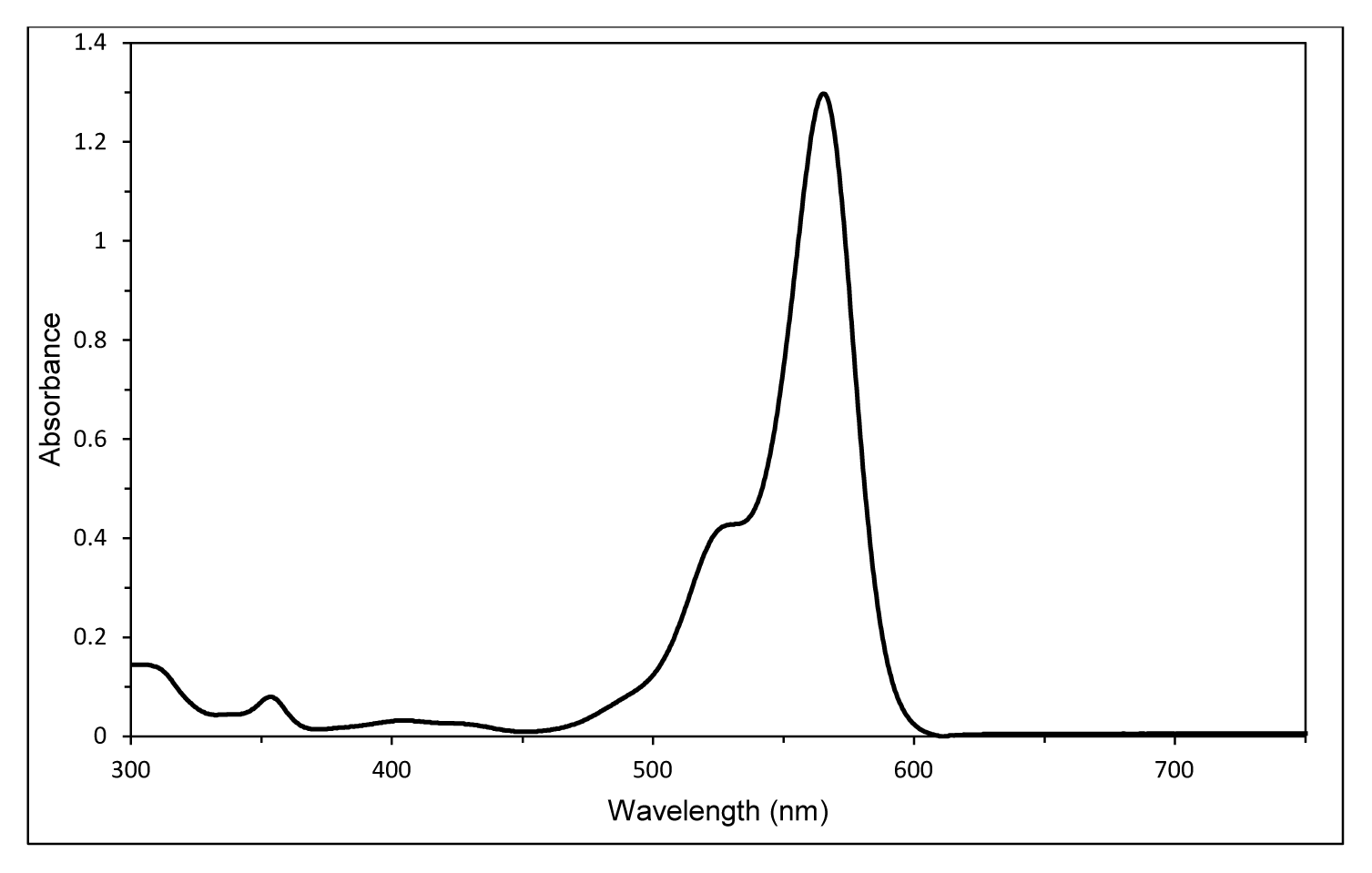

Supplement: Figure S3 — 8.52 × 10^−5^ M SRB in water (max. absorption at 565.5 nm). [file tjc-49-01-118s3.tif]

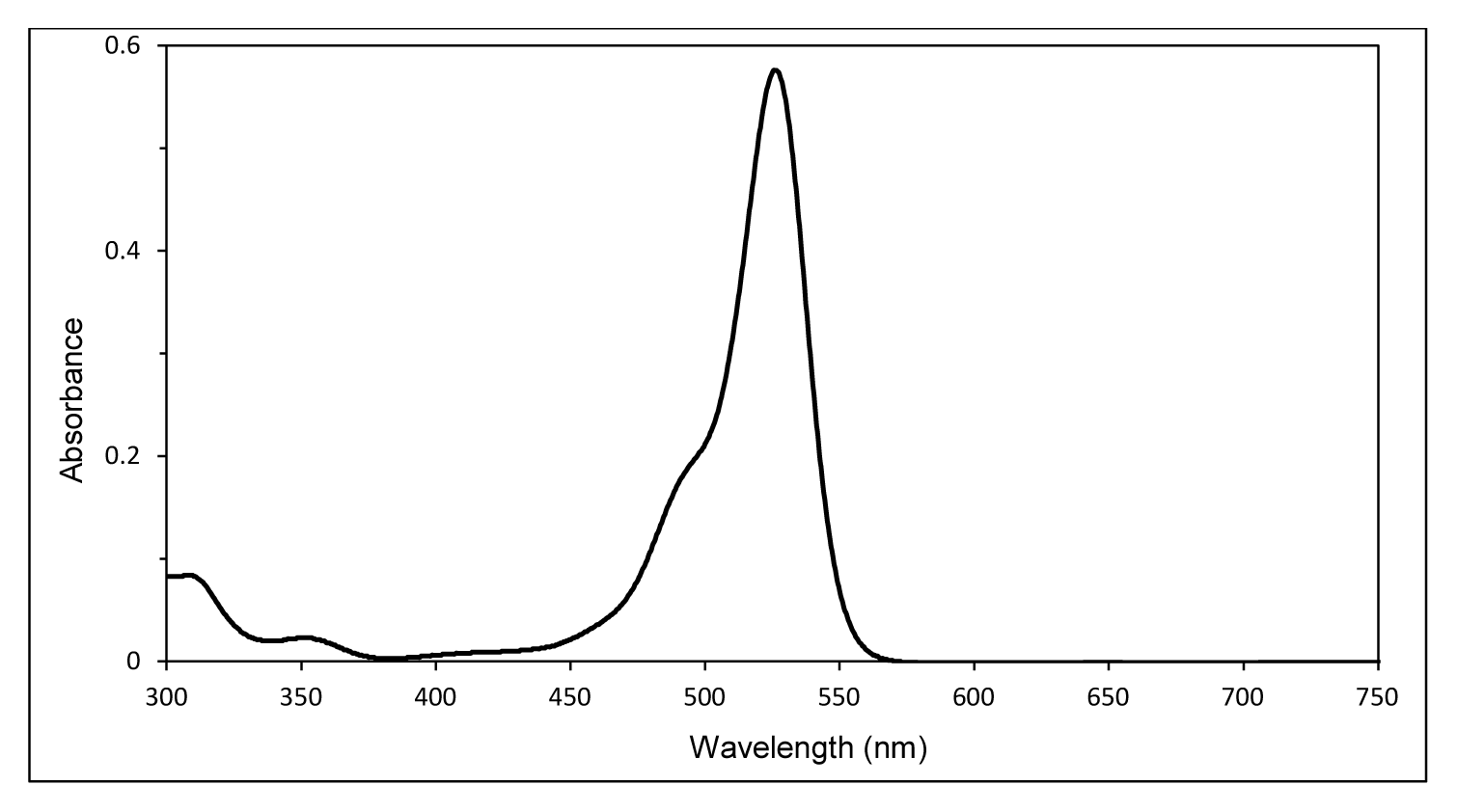

Supplement: Figure S4 — 4.40 × 10^−5^ M ERB in aqueous solution (max. absorption at 526 nm). [file tjc-49-01-118s4.tif]

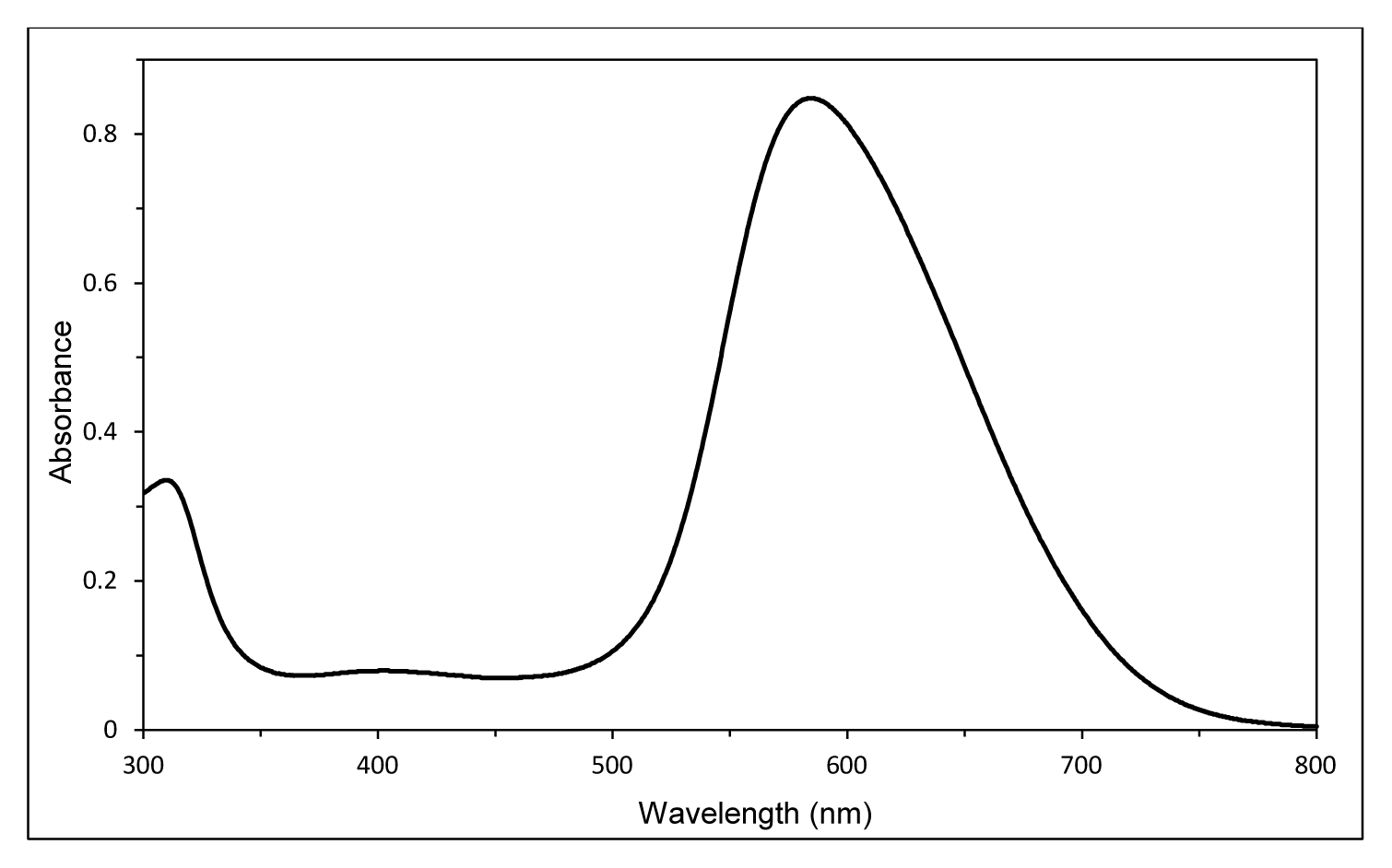

Supplement: Figure S5 — 5.77 × 10^−5^ M BRB in aqueous solution (max. absorption at 584 nm). [file tjc-49-01-118s5.tif]

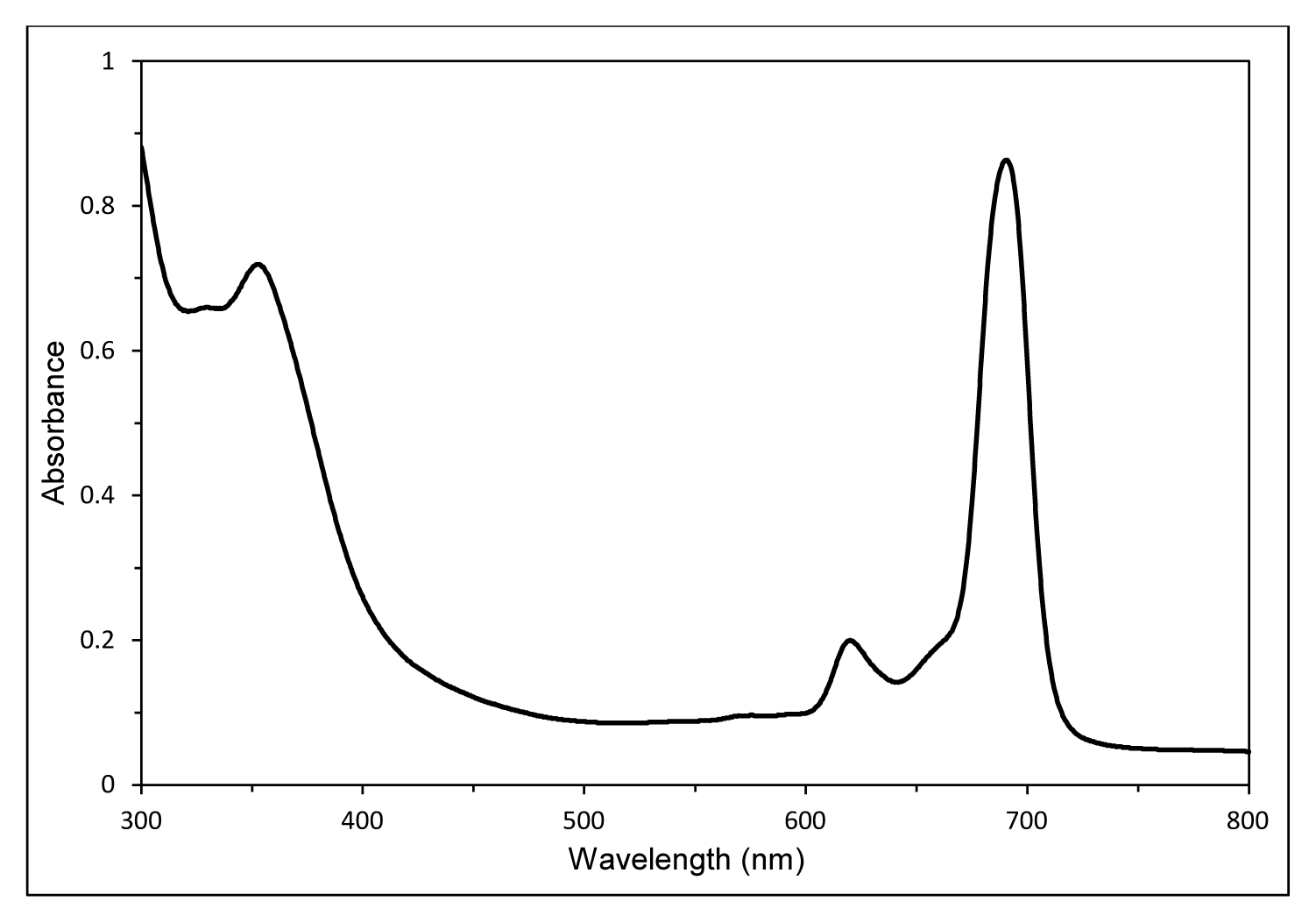

Supplement: Figure S6 — 5.70 × 10^−4^ M compound 2 (Q band at 690 nm, shoulder at 620 nm, and B band at 354 nm). [file tjc-49-01-118s6.tif]

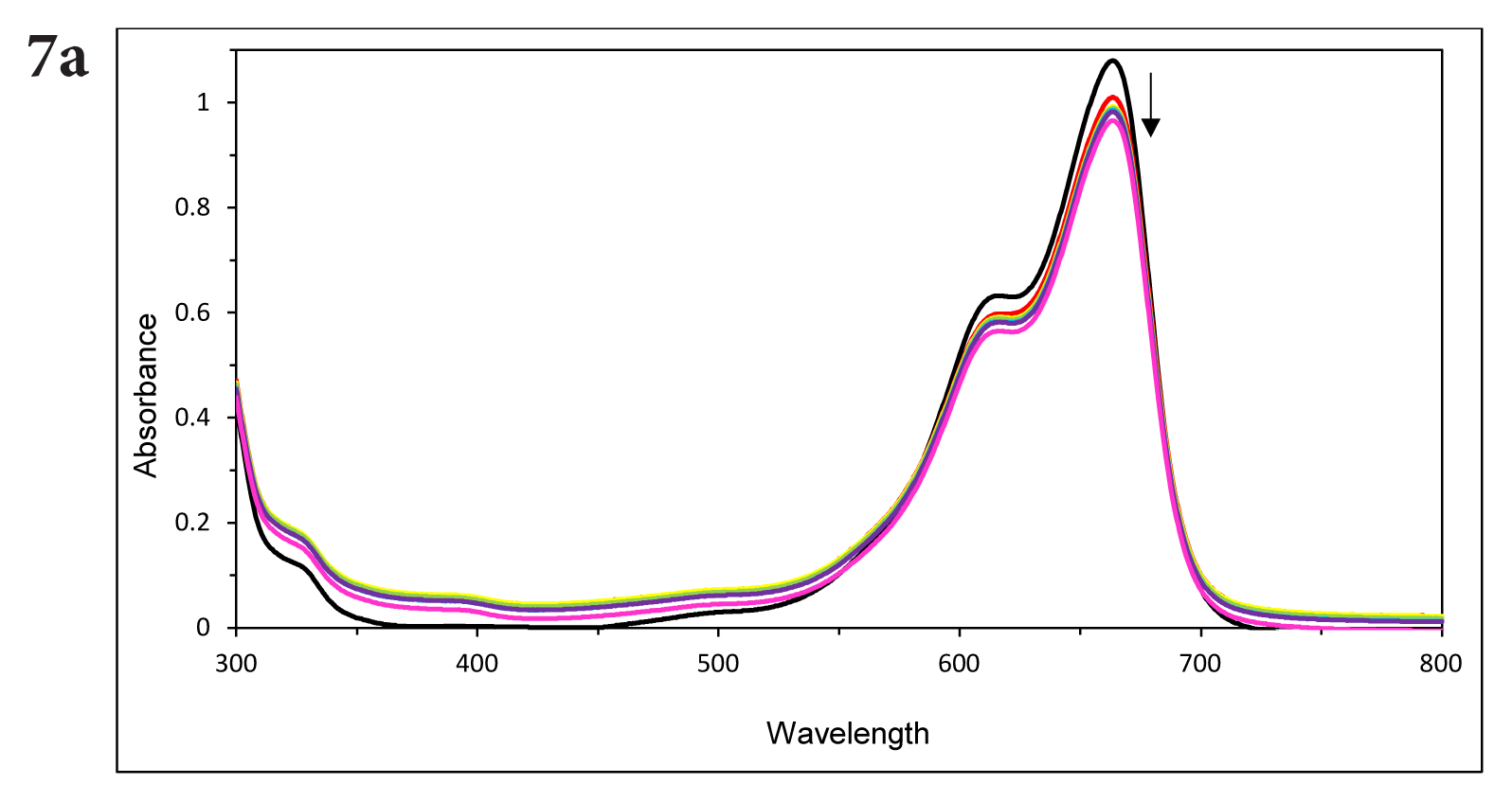

Supplement: Figure S7a — Zero-order derivative spectrum of 1.14 × 10^−5^ M MB dye under 30-s irradiation. [file tjc-49-01-118s7a.tif]

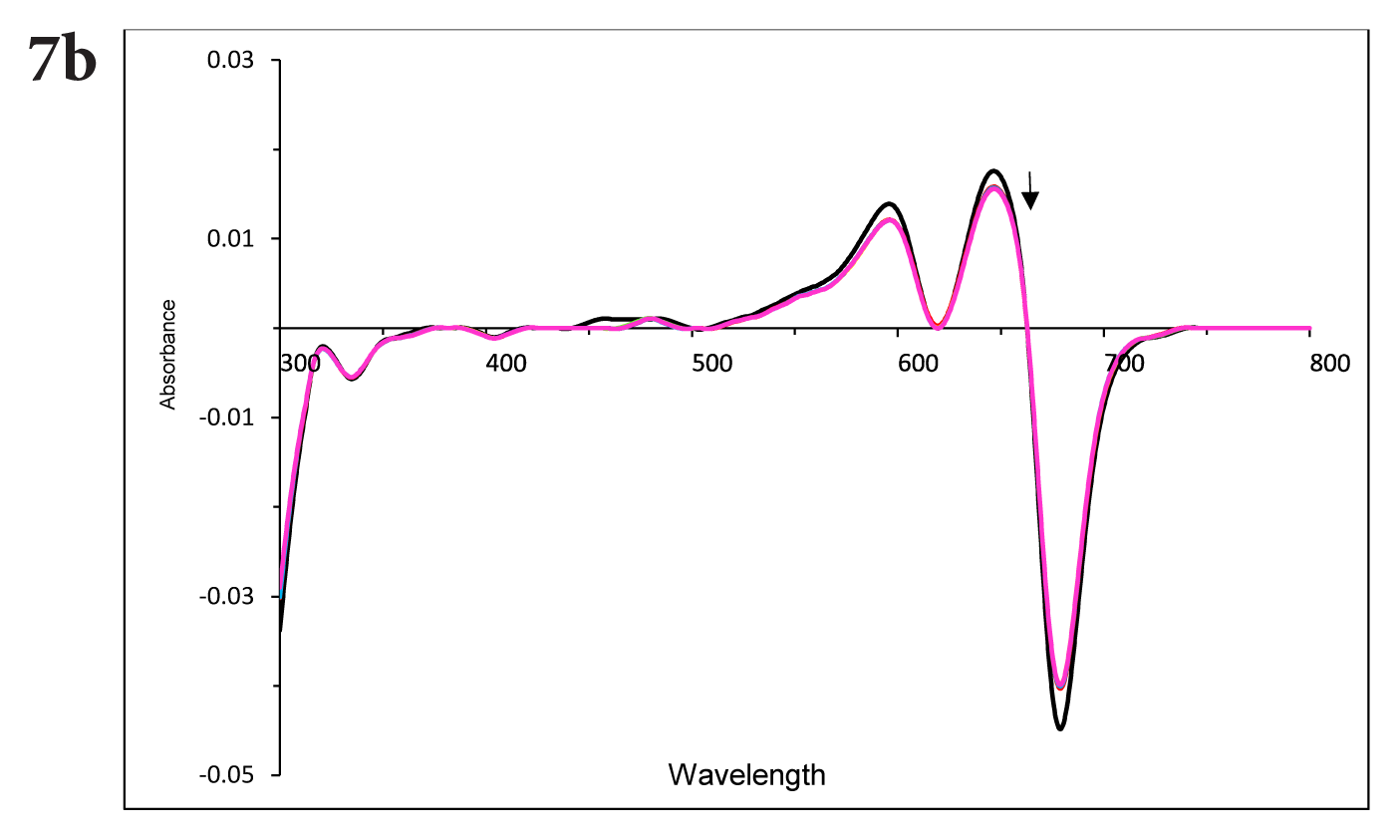

Supplement: Figure S7b — First-order derivative spectrum of 1.14 × 10^−5^ M MB dye under 30-s irradiation. [file tjc-49-01-118s7b.tif]

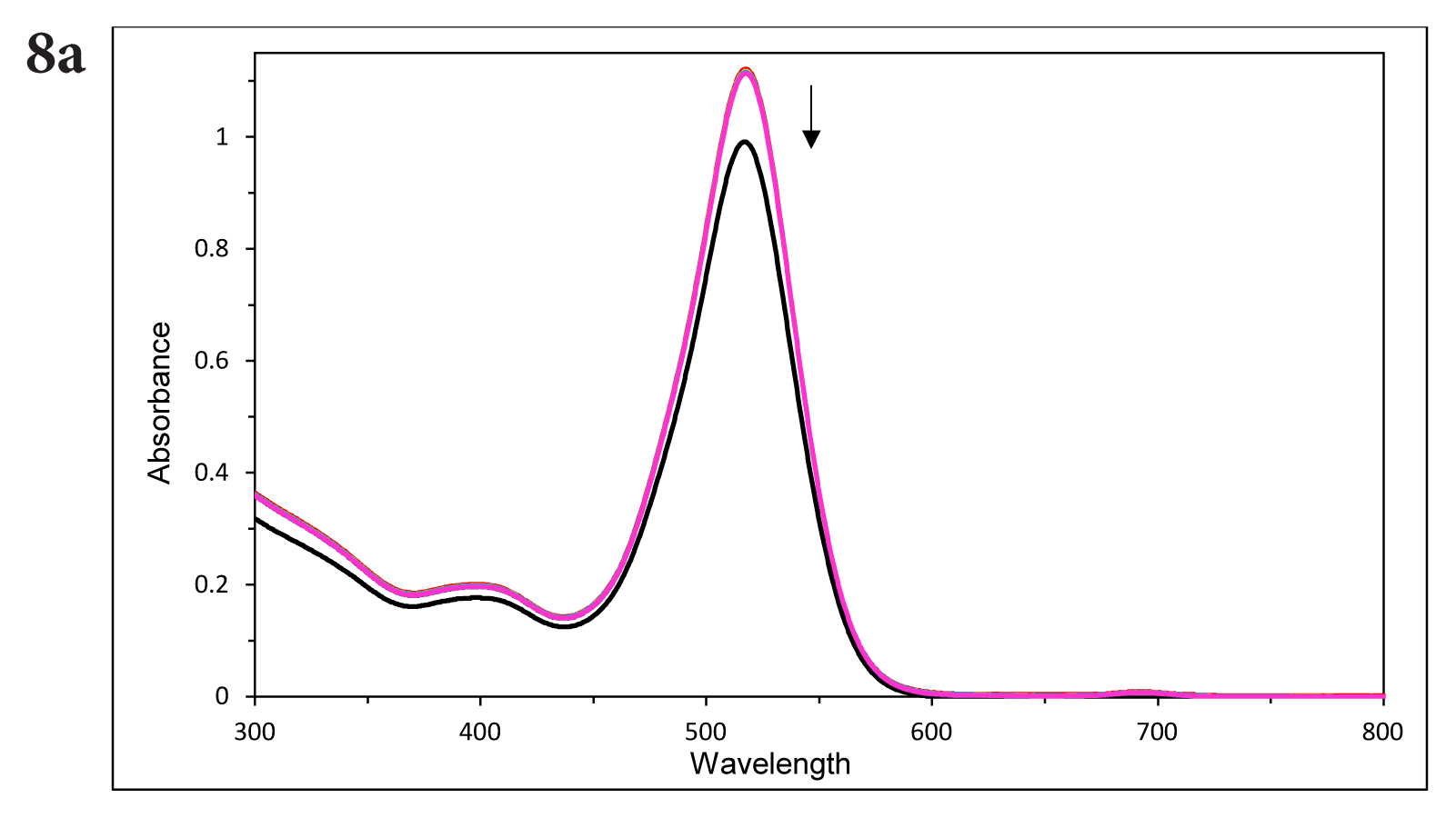

Supplement: Figure S8a — Zero-order derivative spectrum of 2.18 × 10^−5^ M EB dye under 30-s irradiation. [file tjc-49-01-118s8a.tif]

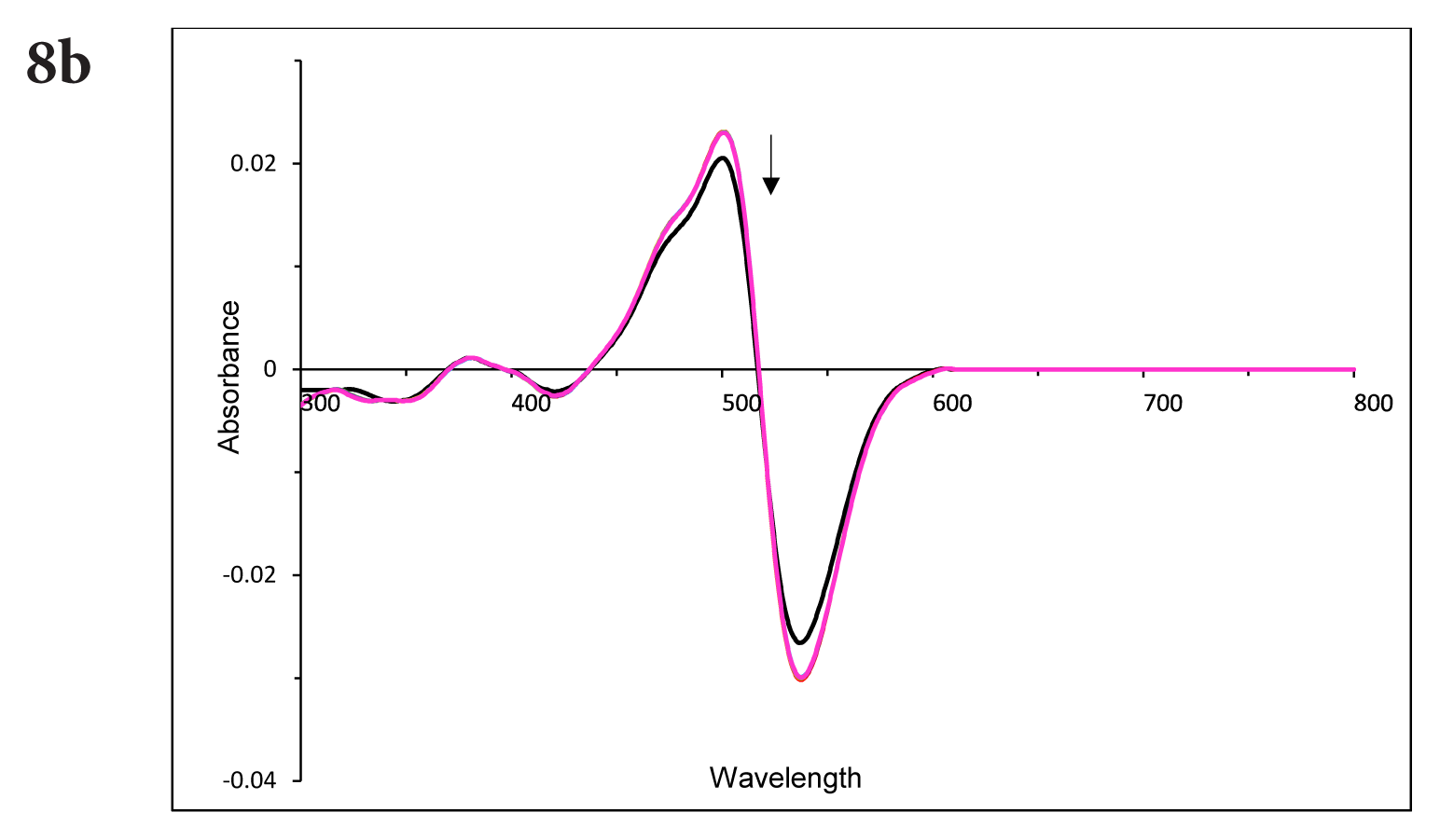

Supplement: Figure S8b — First-order derivative spectrum of 2.18 × 10^−5^ M EB dye under 30-s irradiation. [file tjc-49-01-118s8b.tif]

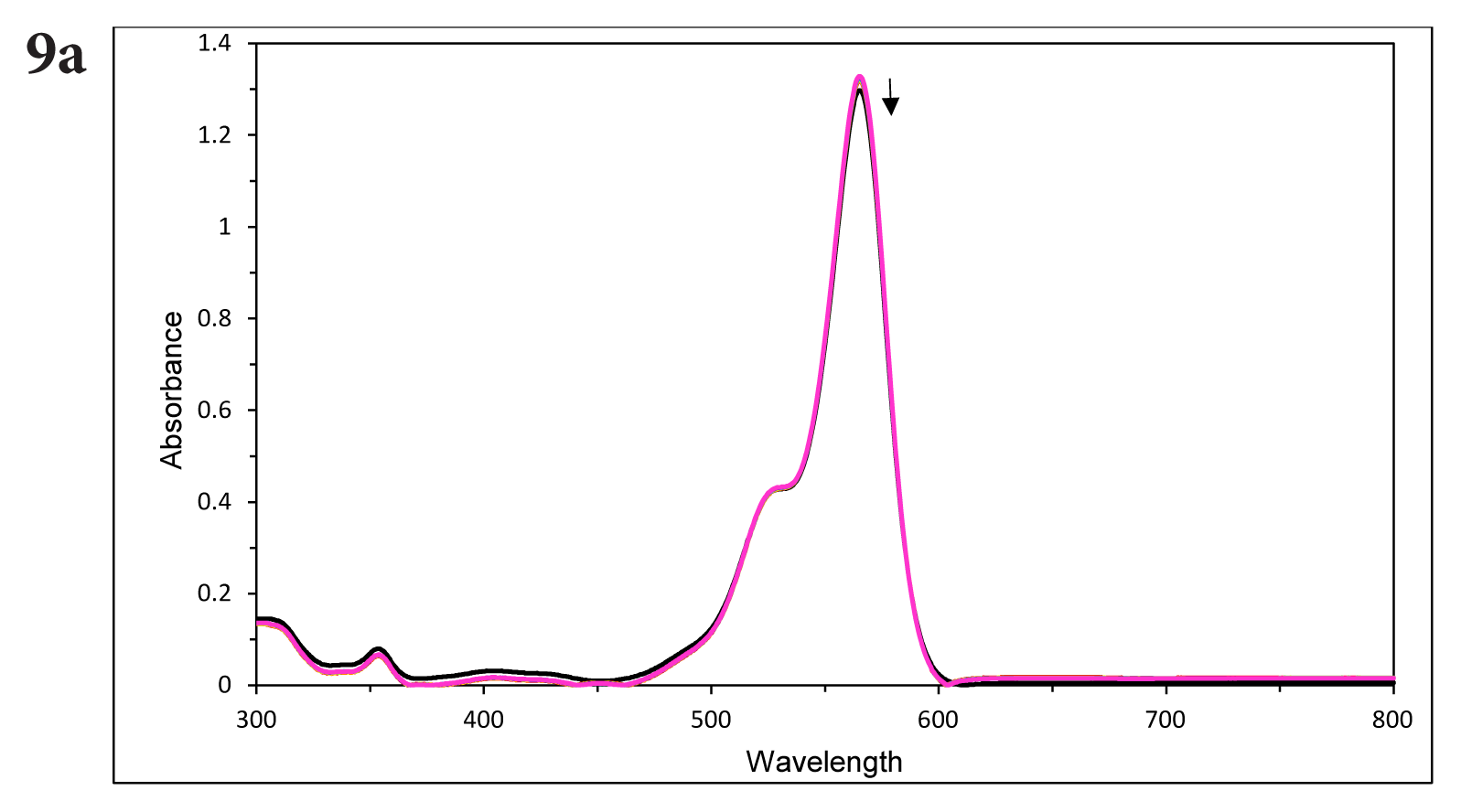

Supplement: Figure S9a — Zero-order derivative spectrum of 8.52 × 10^−5^ M SRB dye under 30-s irradiation. [file tjc-49-01-118s9a.tif]

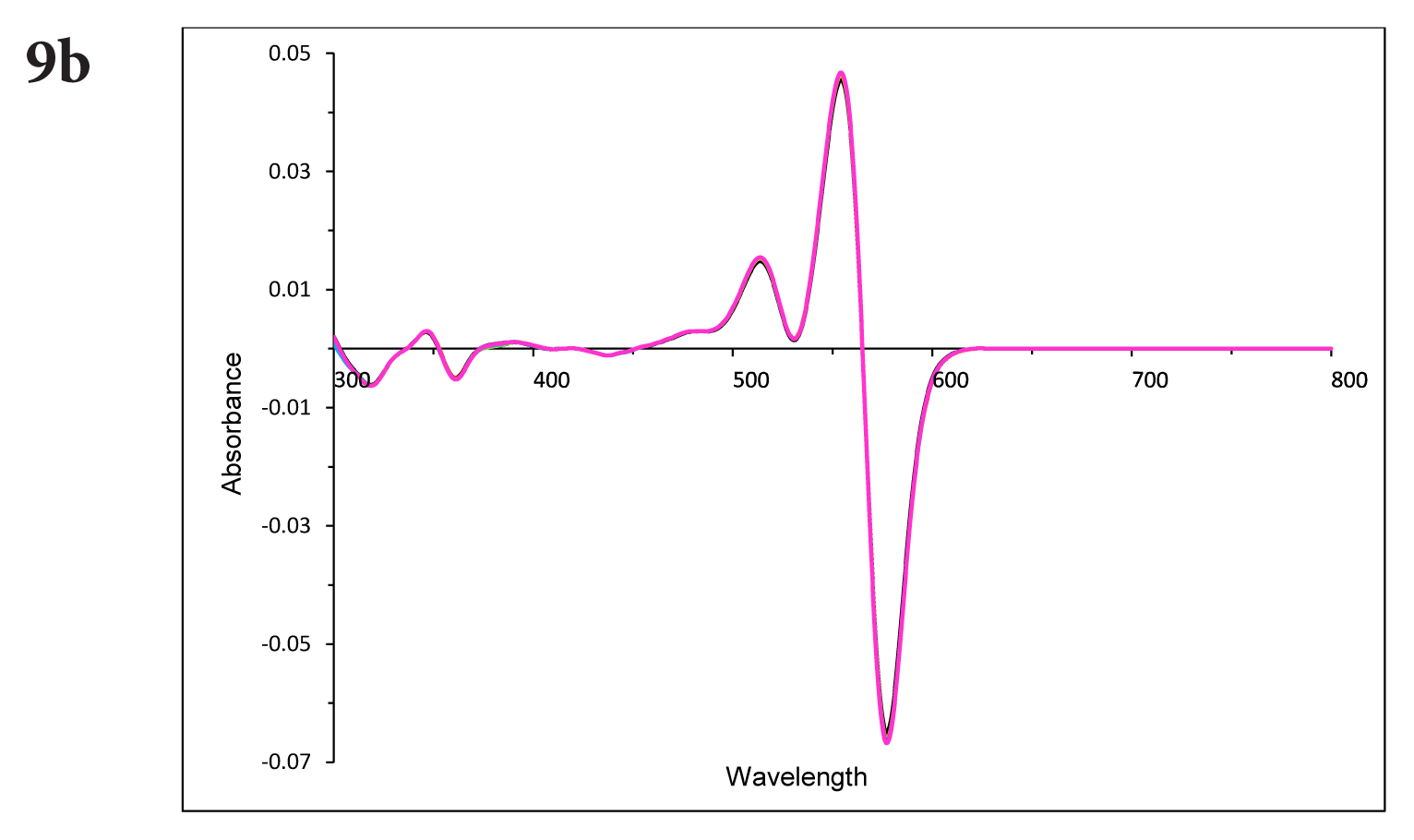

Supplement: Figure S9b — First-order derivative spectrum of 8.52 × 10^−5^ M SRB dye under 30-s irradiation. [file tjc-49-01-118s9b.tif]

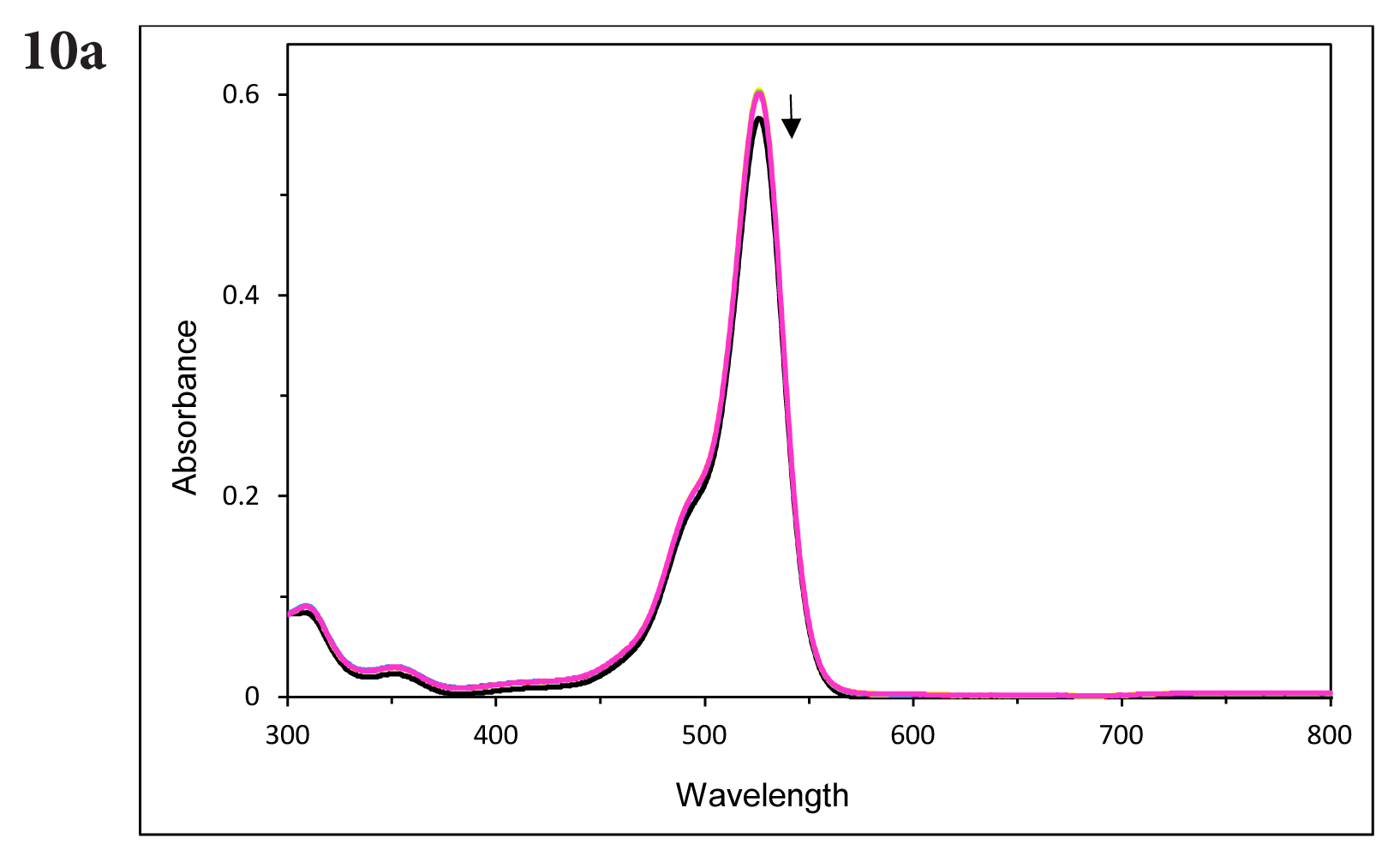

Supplement: Figure S10a — Zero-order derivative spectrum of 4.41 × 10^−5^ M ERB dye under 30-s irradiation. [file tjc-49-01-118s10a.tif]

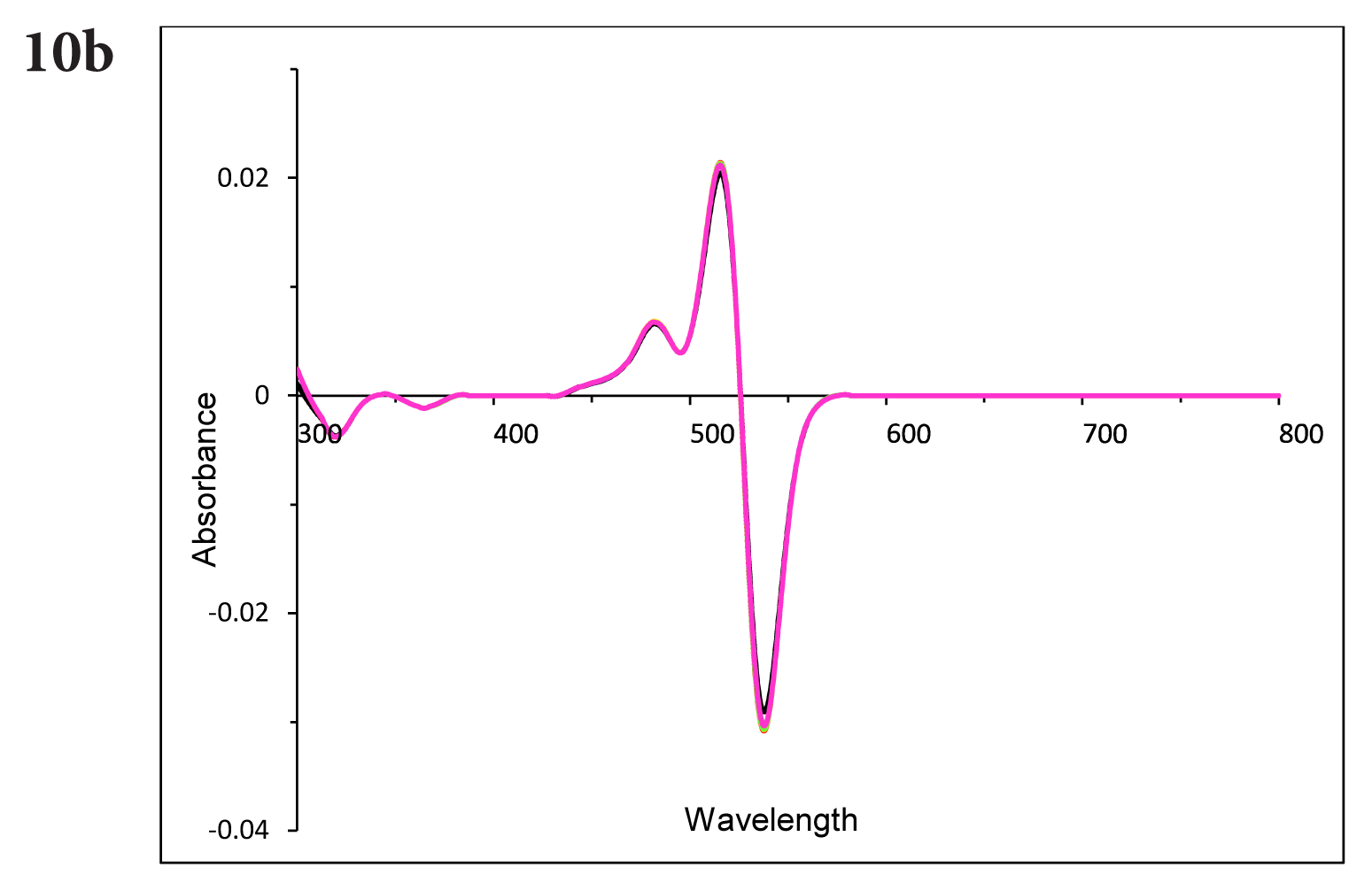

Supplement: Figure S10b — First-order derivative spectrum of 4.41 × 10^−5^ M ERB dye under 30-s irradiation. [file tjc-49-01-118s10b.tif]

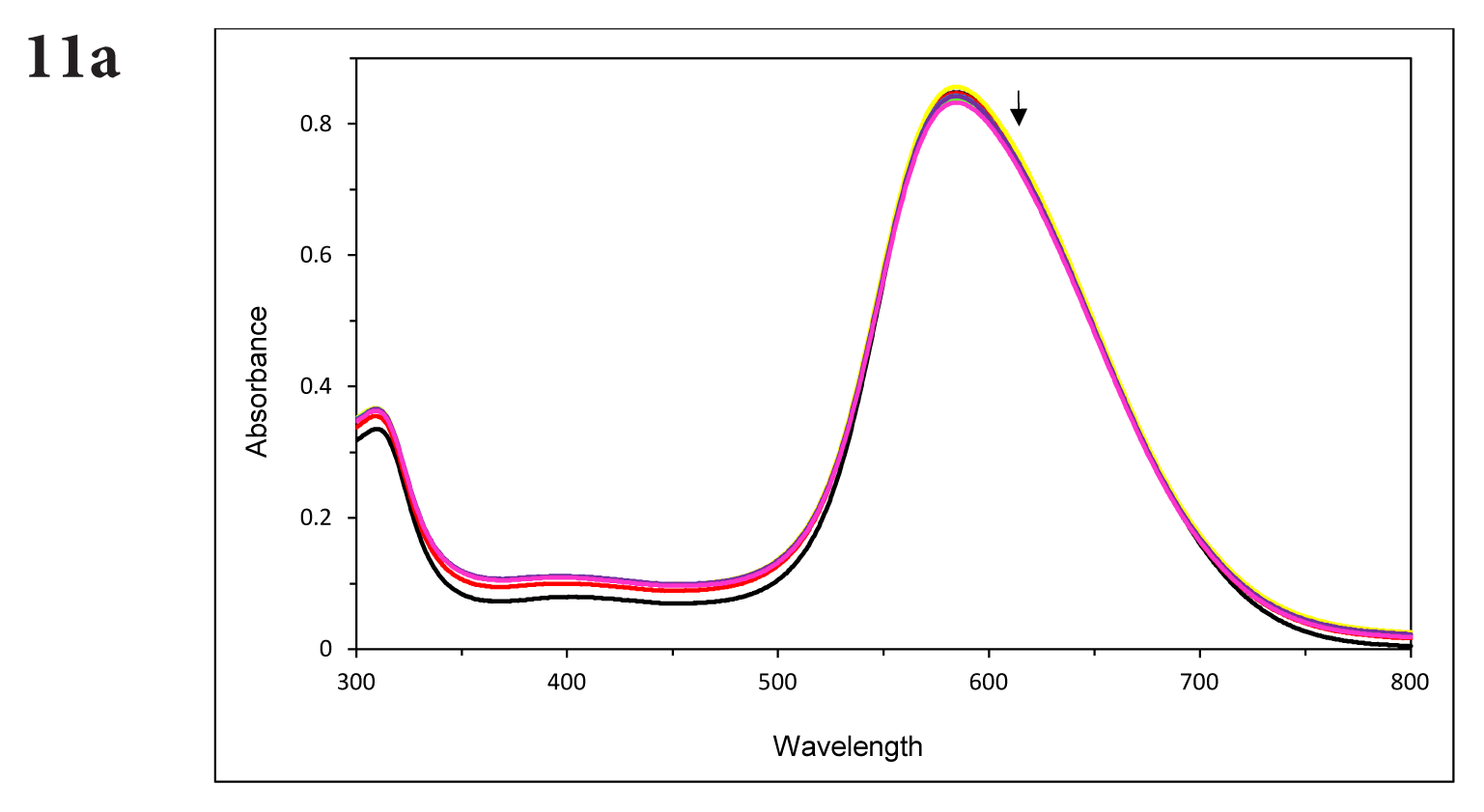

Supplement: Figure S11a — Zero-order derivative spectrum of 5.77 × 10^−5^ M BRB dye under 30-s irradiation. [file tjc-49-01-118s11a.tif]

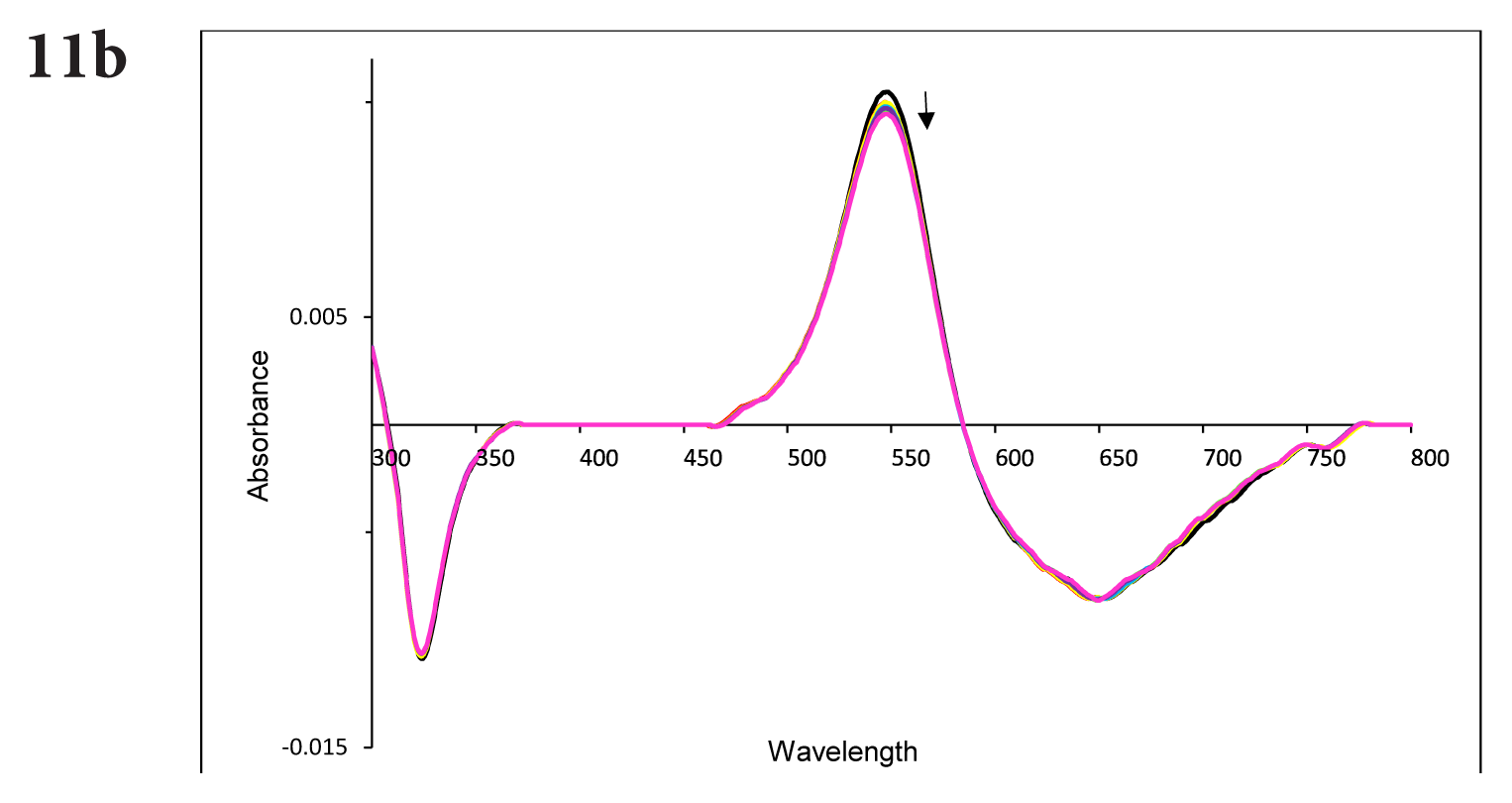

Supplement: Figure S11b — First-order derivative spectrum of 5.77 × 10^−5^ M BRB dye under 30-s irradiation. [file tjc-49-01-118s11b.tif]

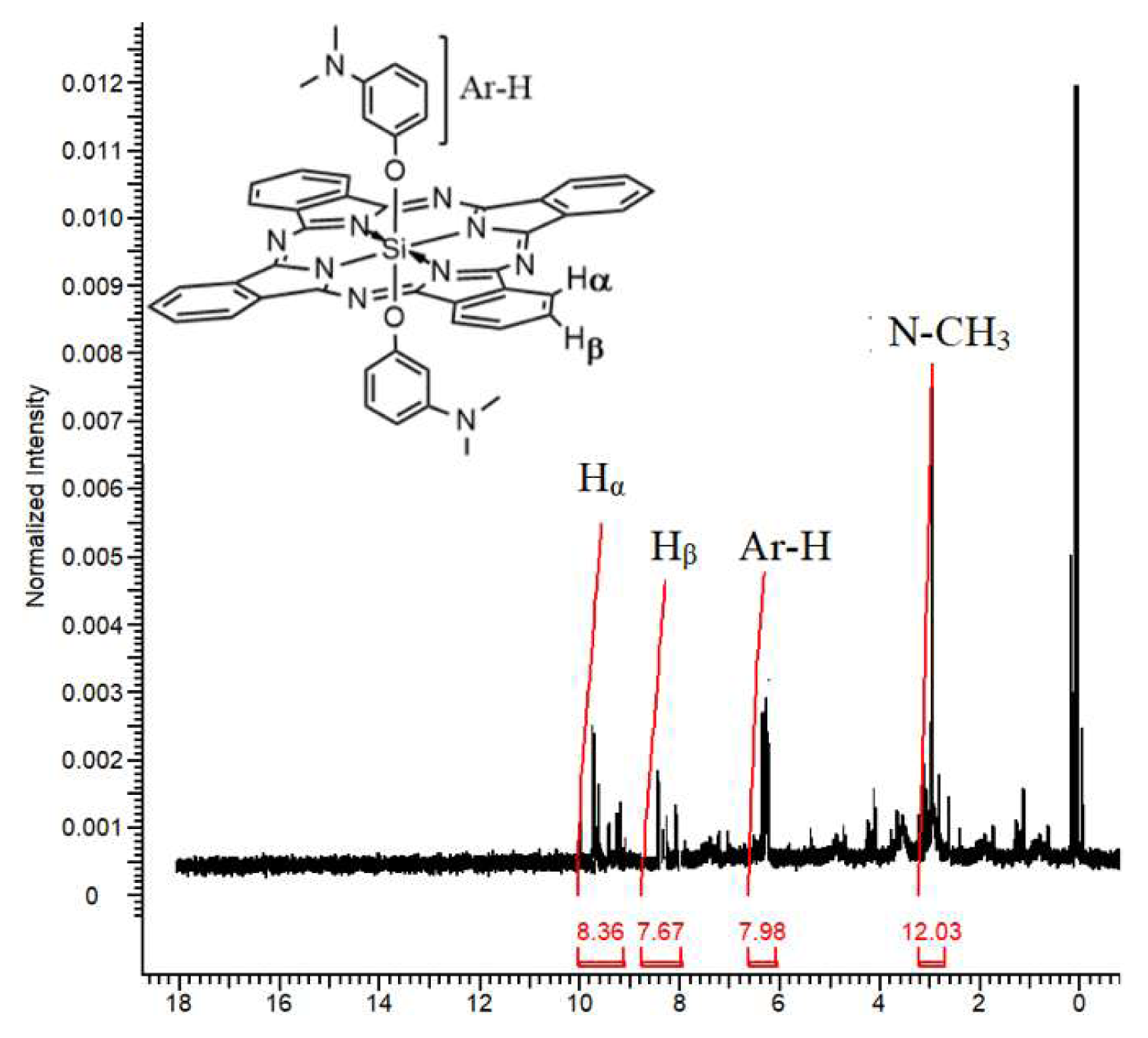

Supplement: Figure S12 — 1H NMR spectrum of compound 1 (DMSO-d6). [file tjc-49-01-118s12.tif]

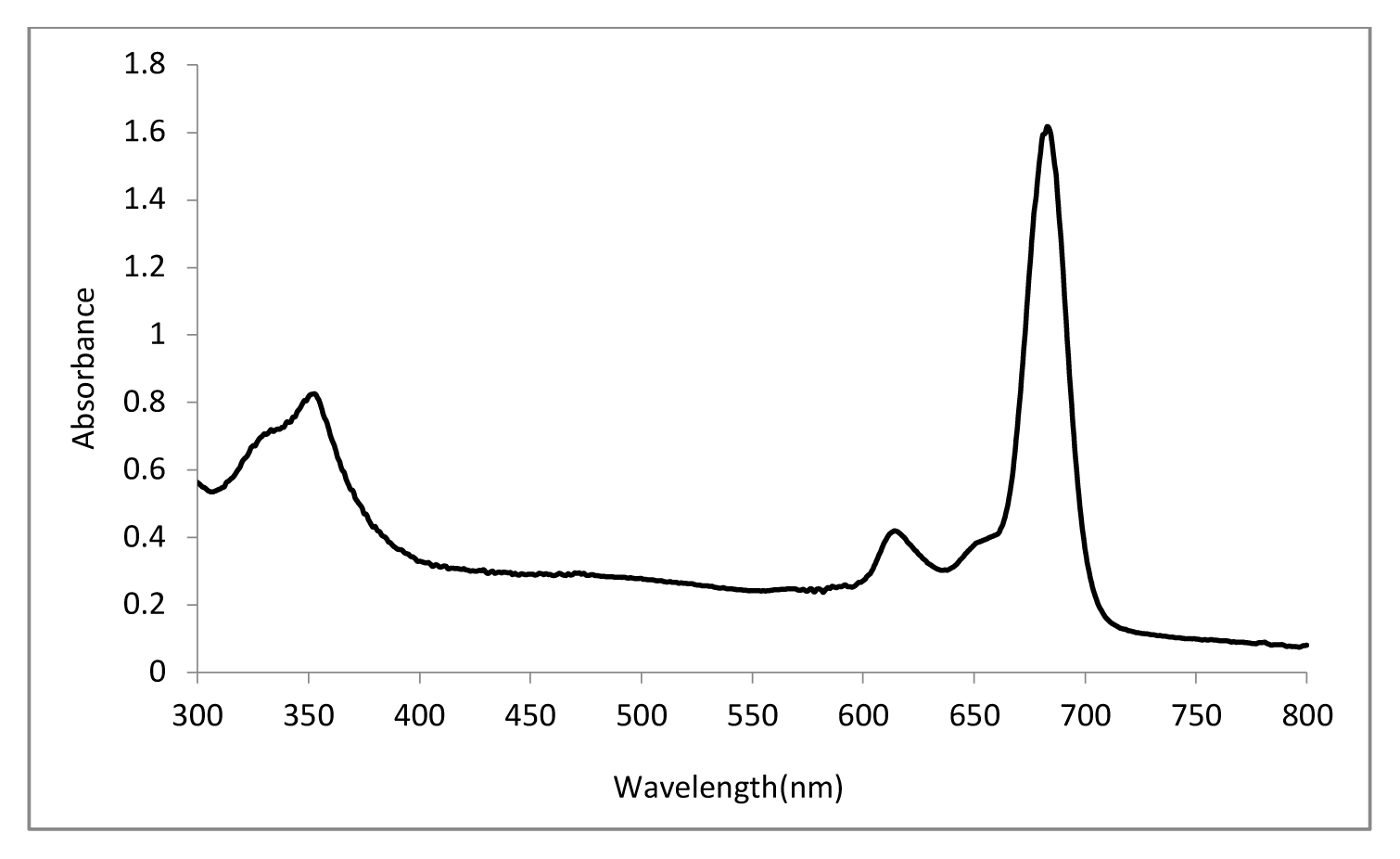

Supplement: Figure S13 — UV-vis spectrum of 4.82 × 10^−5^ M compound 1 in THF (Q band at 683 nm, shoulder at 614 nm, and B band at 353 nm). [file tjc-49-01-118s13.tif]

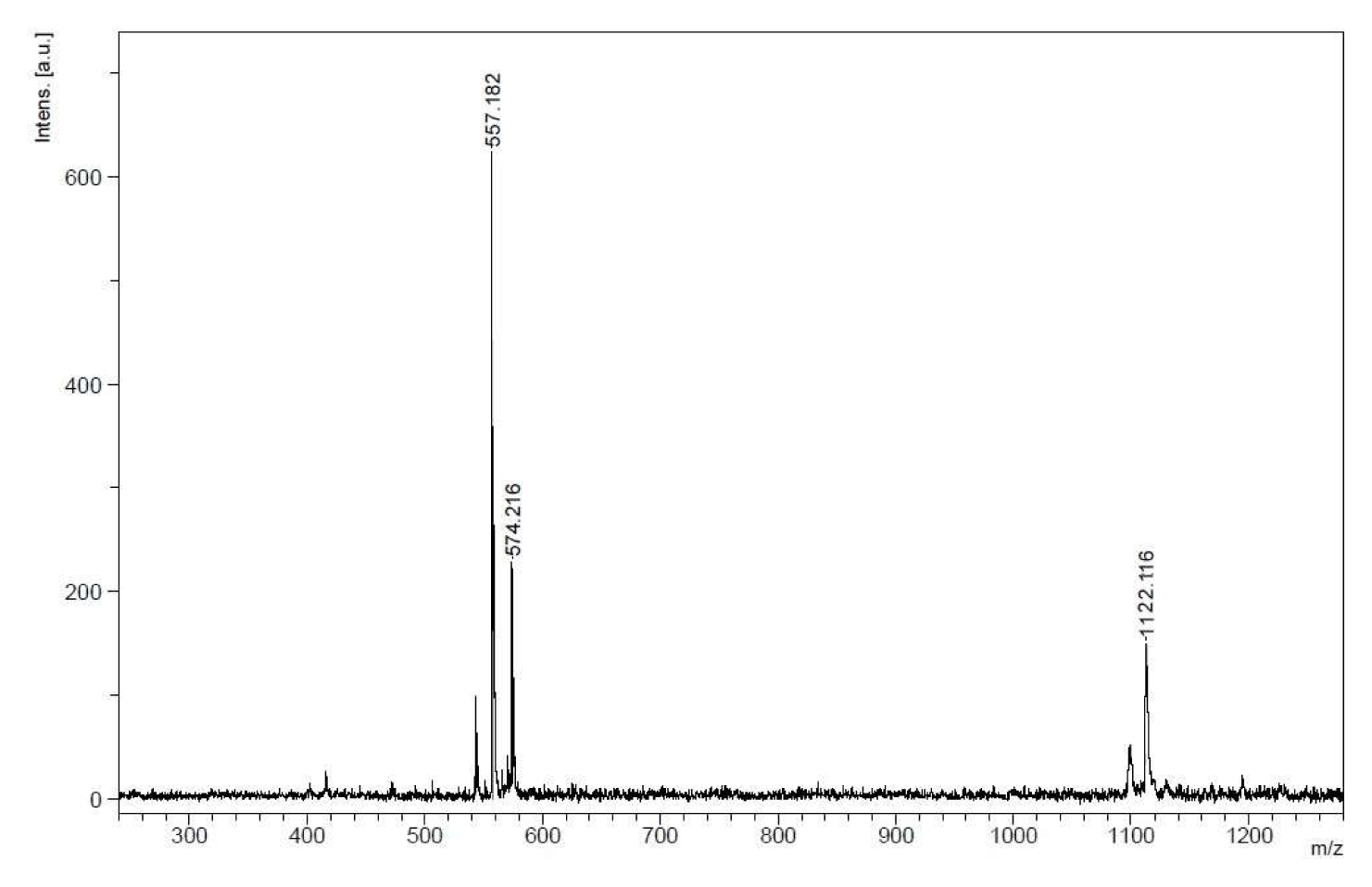

Supplement: Figure S14 — MALDI-TOF spectrum of compound 1. [file tjc-49-01-118s14.tif]

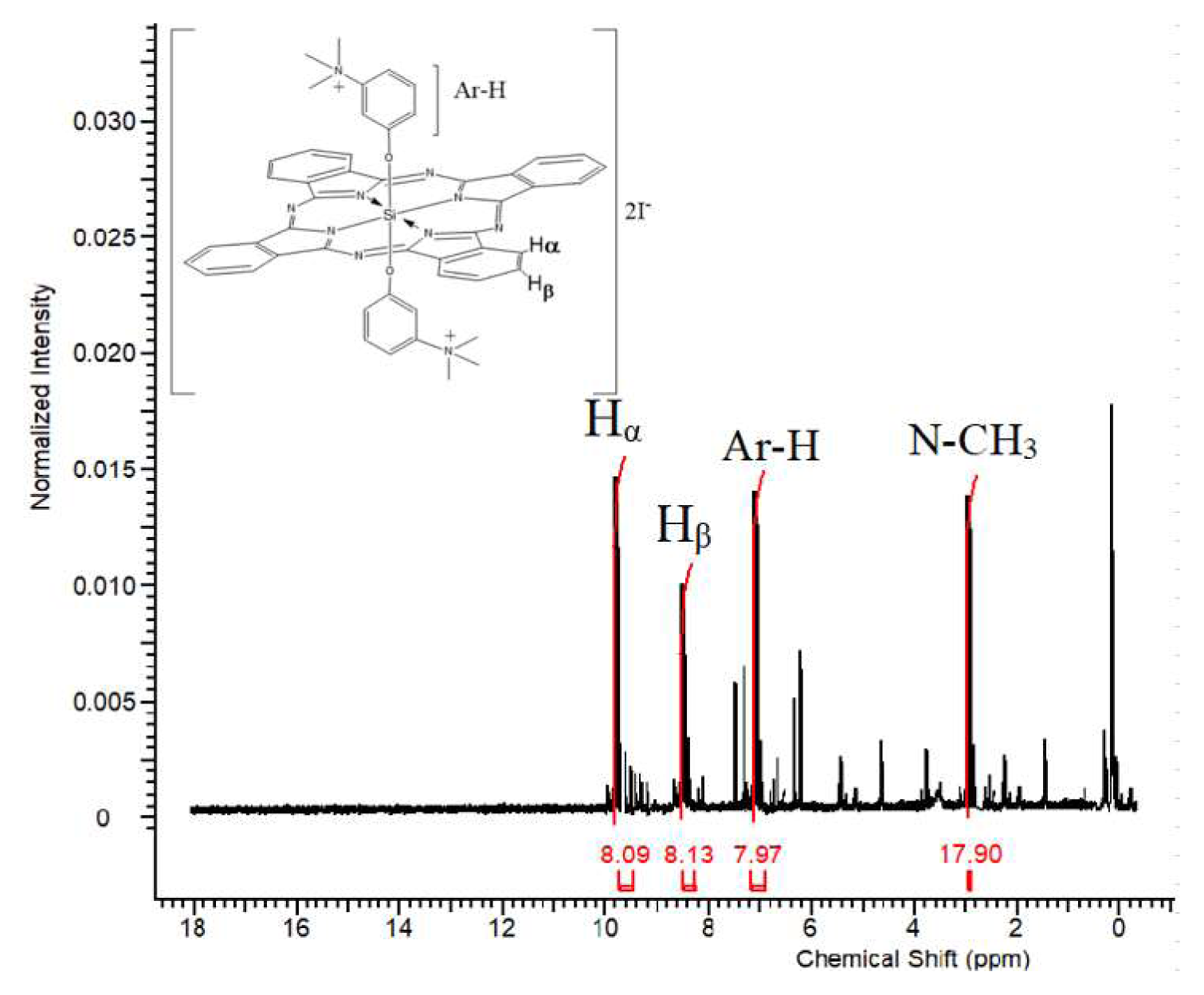

Supplement: Figure S15 — 1H NMR spectrum of compound 2 (DMSO-d6). [file tjc-49-01-118s15.tif]

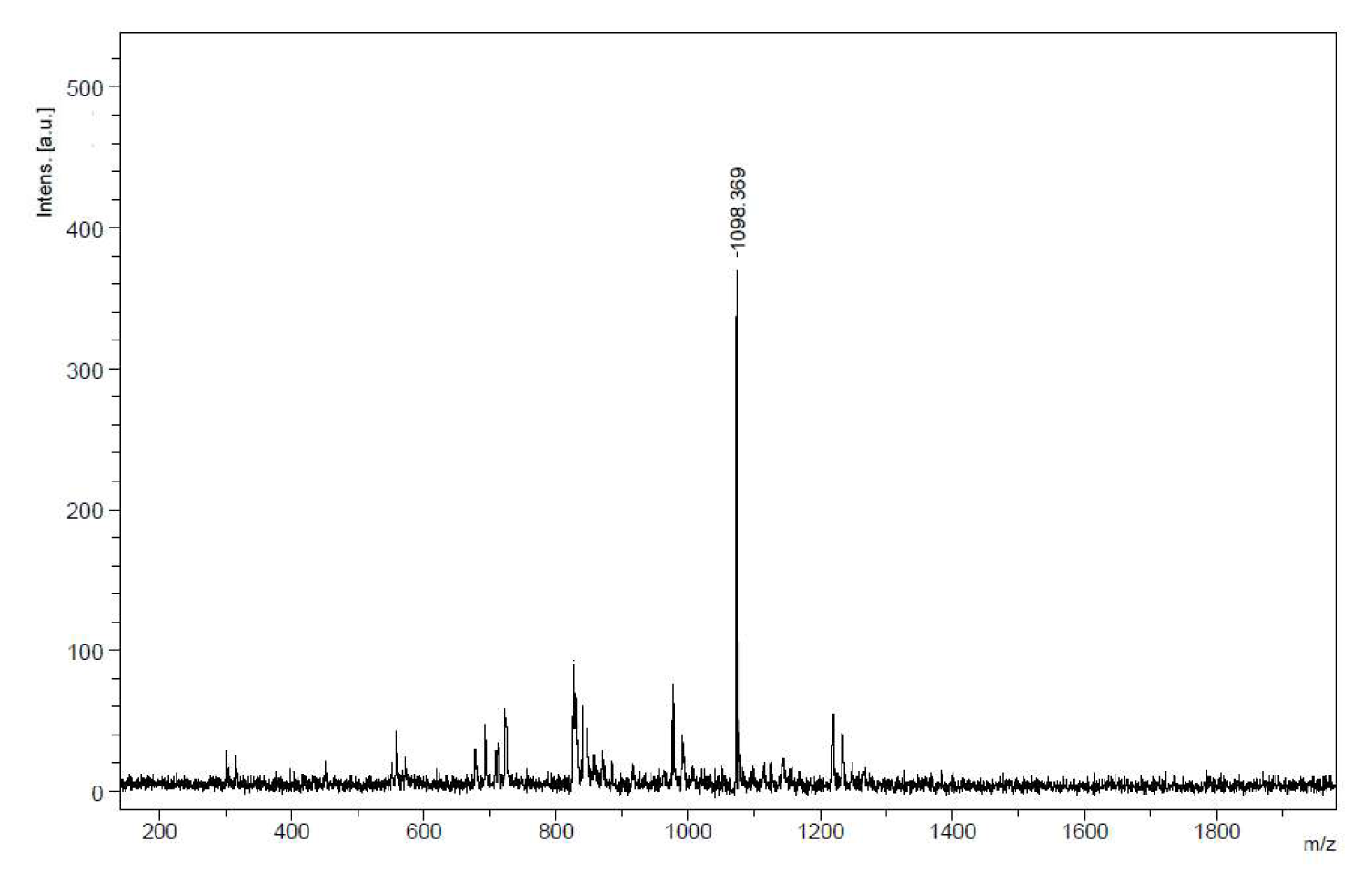

Supplement: Figure S16 — MALDI-TOF spectrum of compound 2. [file tjc-49-01-118s16.tif]
